# Global Hypomethylation in Cell-Free DNA Enables Noninvasive Colorectal Cancer Screening: Results from a Retrospective Validation Study

**DOI:** 10.34133/csbj.0181

**Published:** 2026-08-28

**Authors:** Shahdeep Kaur, Shefali Lathwal, Smita Agrawal, Tina Mahmoudi, Shalini Gupta

**Affiliations:** ^1^ Asima Health Inc., Kitchener, ON N2G 4Z5, Canada.; ^2^ 101GenAI Inc., San Jose, CA 94123, USA.

## Abstract

Global DNA hypomethylation is a defining hallmark of colorectal cancer (CRC) but is poorly captured by existing cell-free DNA (cfDNA) technologies, which typically interrogate only a fraction of CpG sites and are biased toward CpG islands. Asima Rev is a rapid, label-free, whole-genome electrical impedance cfDNA assay previously characterized across 216 clinical samples spanning 15 cancer types, from which a diagnostic threshold was established. The assay differentiates healthy individuals from those with cancer by measuring cfDNA aggregation patterns associated with methylation state, enabling functional detection of genome-wide hypomethylation. In this study, this prespecified threshold (adjusted to the present electrode geometry via cell constant normalization) is applied without modification to an independently collected cohort of 46 treatment-naïve patients with CRC and 33 controls, constituting a disease-specific retrospective validation of the assay. Only 4 samples overlapped between the 2 cohorts; all remaining samples were independently collected, processed, and analyzed. Asima Rev achieved 95.65% sensitivity (95% confidence interval, 85.47% to 99.23%) and 93.94% specificity (95% confidence interval, 80.39% to 98.92%), with longitudinal monitoring in 6 patients fully concordant with clinical outcomes. Interrogation of 11 public methylation array datasets showed that whole-array analyses underestimate global changes. Restricting analyses to OpenSea regions, where cfDNA is enriched, however, revealed patterns consistent with Asima Rev and a significant 5.8% global hypomethylation in CRC tissue compared to adjacent normal tissue. Hypomethylation was not observed in immune cell genomic DNA, a major contributor to cfDNA, supporting a predominantly tumor-derived contribution to the observed cfDNA signal. Together, these results demonstrate that Asima Rev captures a cfDNA signal consistent with true global methylation loss and outperforms locus-specific assays by measuring structural consequences of pan-genomic epigenetic alterations.

## Introduction

Colorectal cancer (CRC) is the third most common cancer globally and the second leading cause of cancer-related mortality, accounting for nearly 1 million deaths annually [1]. Early detection and timely intervention significantly improve survival, with 5-year rates exceeding 70% for locoregional disease [2]. However, population-level screening adherence remains suboptimal, particularly in low-resource or decentralized settings. Despite extensive public health campaigns, only about 60% to 65% of eligible individuals in the United States undergo routine CRC screening [3], with even lower adherence in underserved populations. This gap underscores the need for noninvasive, scalable diagnostic tools that are clinically robust, operationally simple, and economically viable.

Epigenetic alterations, particularly DNA methylation, are a hallmark of many cancers, including CRC [4]. Global hypomethylation was first described in CRC tissues more than 4 decades ago using genome-wide biochemical assays such as enzymatic digestion, followed by liquid chromatography [5], or methyl-cytosine-sensitive restriction endonucleases, followed by gel electrophoresis [6]. While crude by modern standards, these methods truly captured whole-genome methylation loss. Newer technologies such as sequencing, methylation arrays, and polymerase-chain-reaction-based panels have confirmed that CRC tissues exhibit widespread global hypomethylation along with localized hypermethylation at promoter CpG islands [4,7] and have primarily focused on site-specific changes [8]. CRC exhibits complex locus-specific methylation alterations, including paradoxical hypermethylation-associated transcriptional activation of Polycomb-targeted genes, further highlighting the extensive epigenetic remodeling that accompanies colorectal tumorigenesis [9]. Distinct epigenetic patterns also define CRC subtypes, including the CpG island methylator phenotype (CIMP) characterized by vast hypermethylation of promoter CpG island sites [10], microsatellite instability (MSI) driven by methylation-mediated mismatch repair silencing [11], and consensus molecular subtypes (CMS1 to CMS4) [12], each characterized by specific epigenetic and genomic alterations. This increased resolution has shifted clinical applications toward promoter-targeted methylation assays [4].

Most modern methylation technologies interrogate only a small fraction of the genome. Methylation arrays typically cover 450,000 to 850,000 CpGs (~1.6% to 3.0% of the approximately 28 million CpG sites present in the human methylome) [13], while sequencing-based assays often rely on restricted panels to maintain signal sensitivity and lower the cost of whole-genome bisulfite sequencing. These single-nucleotide or short-region measurements—akin to examining individual “beads” on a DNA necklace—enable detailed molecular profiling but require complex workflows, specialized personnel and reagents, and costly instrumentation. As a result, their broad population deployment remains challenging.

Cell-free DNA (cfDNA) has emerged as a minimally invasive analyte for CRC screening, prognosis, and disease monitoring [14]. However, despite global hypomethylation being one of the earliest and most fundamental epigenetic hallmarks of CRC tissues [5,6], it remains underexplored in cfDNA. Technical barriers including high DNA input requirements for arrays (~200 to 250 ng per test) [15,16], sensitivity limitations, and the predominance of clinical interest in targeted gene panels have restricted cfDNA studies largely to locus-specific assays using sequencing or polymerase-chain-reaction-based methods. Some studies have used methylation of long interspersed nucleotide element-1 (LINE-1) as a proxy measure of global DNA methylation level and observed hypomethylation in both tissue and plasma of CRC patients [17]. However, genome-wide studies including those with methylation array datasets for cfDNA remain scarce, limited to small sample numbers [18] or pooled samples [19,20]. As a result, no global hypomethylation assay has translated into cfDNA diagnostics despite extensive tissue evidence. However, global hypomethylation remains an appealing biomarker if it can be measured rapidly and inexpensively, as it offers a robust, heterogeneity-agnostic signal capable of segregating healthy and cancer samples at scale [21].

Our group recently demonstrated a novel cfDNA assay called Asima Rev that analyzes the global structure of cfDNA—the “necklace”—rather than individual nucleotides, by electrically measuring cfDNA aggregation as a surrogate biomarker for genome-wide methylation changes during cancer [22]. Global methylation changes are known to alter cfDNA’s physicochemical properties, including charge density, flexibility, conformation, and higher-order structure, which together influence its electrical impedance signature. By measuring these properties in a rapid, label-less, and amplification-free manner on a chip, Asima Rev detects tumor-associated methylation shifts without the complexity of sequencing workflows and with potential pan-cancer applicability. In the previous study, the assay was characterized across 216 clinical samples spanning 15 cancer types. A diagnostic threshold was established, and pan-cancer utility was demonstrated by exploiting global methylation alterations conserved across malignancies. In the present study, we apply this prespecified threshold—scaled to the present electrode geometry via cell constant normalization—to an independently collected cohort of 79 patients with CRC and healthy controls, constituting the first disease-specific retrospective validation of Asima Rev in a clinically actionable screening context.

Using this framework, we evaluate the performance of Asima Rev for CRC detection and surveillance using archived plasma from treatment-naïve patients with CRC and age-matched healthy controls across all disease stages. Beyond diagnostic performance, we address 4 key questions related to the biological and clinical validity of the assay: (a) whether global hypomethylation in cfDNA is a robust and consistent biomarker for CRC, (b) whether Asima Rev functionally measures this hypomethylation signal, (c) how does its performance compare with existing CRC-targeted methylation assays, and (d) whether immune-cell-derived cfDNA—the dominant cfDNA source in both healthy individuals and patients with cancer—could confound the observed signal. The relationship between the Asima Rev signal and cfDNA hypomethylation was established in our previous pan-cancer study using methylated oligonucleotides [22], and is further supported here through multiple complementary analyses. These include transmission electron microscopy (TEM), which shows methylation-associated differences in aggregation behavior, and concordance between Asima Rev measurements with established methylation proxies such as OpenSea and LINE-1 methylation. To further support this mechanistic framework and address the remaining questions, we analyzed 11 public methylation array datasets (Table S1) generated using Illumina Infinium BeadChip arrays [23,24], one of the most widely used platforms for genome-scale methylation profiling and the basis of large-scale efforts including The Cancer Genome Atlas (TCGA). We additionally examined immune cell genomic DNA (gDNA) methylation patterns under CRC and immune-related conditions as a proxy for the immune-derived fraction of cfDNA, given the limited availability of direct cfDNA methylome datasets. Our analyses support that global hypomethylation is a robust feature of CRC and that Asima Rev’s signal is consistent with the previously established methylation-dependent aggregation mechanism, while the present study primarily evaluates the assay’s diagnostic performance rather than independently establishing the mechanism. We finally illustrate that immune-derived DNA exerts minimal influence on the observed signals, although further validation in larger prospective cohorts remains warranted.

## Materials and Methods

### Materials

The following materials were used: platinum-on-glass planar interdigitated electrodes with 40 symmetrical fingers (20 pairs), each 3.8 mm long and 100 μm wide, with 100-μm edge-to-edge spacing (Micrux Technologies, Spain); sodium hydroxide (NaOH) (Sigma-Aldrich, Canada); MagMax cfDNA isolation kit (A29319, Thermo Fisher Scientific); Hepes (Sigma-Aldrich, Canada); polydimethylsiloxane (PDMS) SYLGARD 184 elastomer kit (Dow, Ellsworth Adhesives, Canada); K2/K3EDTA blood collection tubes (Greiner Bio-One, VWR Canada); 200-mesh-square carbon TEM grids (Electron Microscopy Sciences); UranyLess EM stain (Electron Microscopy Sciences); cryovials (Rose Scientific Ltd.). Buffer was prepared at pH 7.4 in ultrapure deionized Milli-Q water (~18.2 MΩ·cm resistivity) following standard procedures, and experiments were conducted at room temperature (RT; 21 to 23 °C).

### Experimental protocol

#### Plasma collection

A total of 46 double-spun, cell-free frozen plasma vials from treatment-naïve patients with confirmed CRC were obtained through Ontario Tumor Bank (Toronto, ON, Canada; *n* = 24), PrecisionMed BioIVT (Carlsbad, CA, USA; *n* = 18), and the Rajiv Gandhi Cancer Institute and Research Centre (Delhi, India; *n* = 4) (Institutional Review Board-approved studies: 2023-3365-16254-6, 2023-3365-16271-4, and Res/BR/TRB-19/2020/56). The cohort included 24 males and 22 females, aged 45 to 75 years, encompassing all 4 CRC stages (stage I, 6; stage II, 14; stage III, 13; stage IV, 13) (see Table S2 for details). In addition, longitudinal plasma samples collected over a 24-month period were available for 6 PrecisionMed patients (see Table S3 for details). For controls, 33 blood samples were collected from healthy individuals (15 males and 18 females; aged 35 to 75 years) using the same protocol as the cancer samples and after obtaining informed consent (see Table S4 for details). To minimize preanalytical variability across collection sources, standardized procedures were used for blood collection, plasma processing, storage, cfDNA extraction, and impedance measurements. Potential source-dependent effects were subsequently evaluated by comparing differential impedance values across biobanks, where no statistically significant differences were observed (Fig. S1). Peripheral blood (6 ml) was drawn via venous phlebotomy into K2EDTA tubes. Plasma was processed within 4 h of collection (with blood exposure to RT limited to <1 h). Initial centrifugation was performed at 2,000*g* for 10 min at 4 °C, followed by transfer to 15-ml Falcon tubes and a second centrifugation at 3,000*g* for 15 min at 4 °C. The resulting double-spun plasma was aliquoted into 1-ml cryovials to allow a single freeze-thaw and was stored at –80 °C until further processing.

#### cfDNA extraction and quantification

Plasma samples were thawed by transferring them to −20 °C overnight, followed by incubation at RT for 30 min. cfDNA was then extracted using the MagMax Cell-Free DNA Extraction Kit, following a slightly modified protocol. Briefly, the plasma was mixed with the recommended volumes of lysis/binding solution and magnetic beads and then vortexed for 20 min. The mixture was placed on a magnetic stand for 3 to 5 min to separate the cfDNA-bound magnetic beads. Beads were washed twice with the wash solution and twice with 80% ethanol. After air drying, cfDNA was eluted in 20 μl of elution buffer, referred to as the “cfDNA stock”, and frozen at −20 °C until further use. cfDNA concentration in the stock (*C*_stock_) was quantified using both a NanoDrop One spectrophotometer and a Qubit 4 fluorometer (Thermo Fisher Scientific), following the manufacturers’ protocols.

#### cfDNA sample preparation

**Fig. 1.**
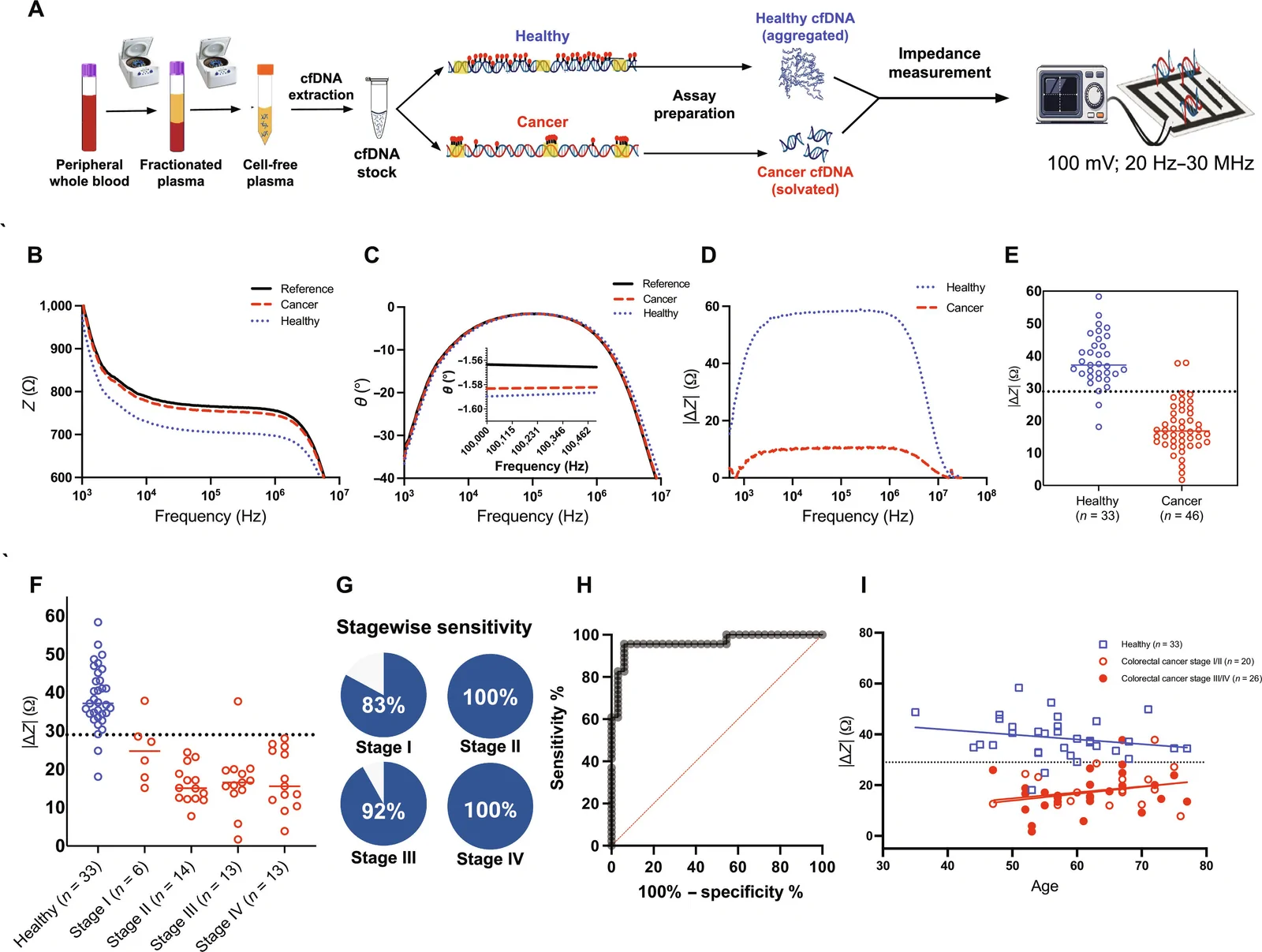
Performance of Asima Rev evaluated in patients with colorectal cancer (CRC) (*n* = 46) and healthy individuals (*n* = 33). (A) Schematic overview of the assay workflow. (B to D) Impedance spectra showing the magnitude (B) and phase angle (C) of impedance, and the magnitude of absolute differential impedance (|Δ*Z*|) (D) as a function of frequency for representative healthy (H19) and cancer (C21) samples. The inset in (C) shows the phase angle variation near 100 kHz. (E and F) Differential impedance values at 100 kHz across healthy (*n* = 33) and all CRC stages (*n* = 46) (E) and stagewise patients with CRC (F). The dotted line indicates the diagnostic threshold at 29 Ω. (G) Pie chart showing stagewise sensitivity for CRC detection. (H) Receiver operating characteristic (ROC) curve demonstrating the assay’s high diagnostic performance (AUC = 0.96). (I) Differential impedance as a function of age of healthy individuals (slope = −0.1894, *P* = 0.2392) and patients with early-stage (slope = 0.1616, *P* = 0.4502) and late-stage (slope = 0.2690, *P* = 0.1739) CRC.

cfDNA stock was thawed at RT and 1 μl was added to 49 μl of 15 mM Hepes buffer (pH 7.4). The sample was then mixed vigorously and stored at 4 °C for 4 to 5 h, followed by equilibration at RT prior to impedance measurements. No concentration normalization was applied across samples because cfDNA concentration did not materially affect discrimination between healthy and CRC samples at the cohort level, as demonstrated previously [20] and confirmed in the present cohort (Fig. 1E and Tables S2 and S4). A reference solution containing 2% (v/v) elution buffer in Hepes was also freshly prepared and used for baselining the impedance measurements.

#### Impedance measurement

A PDMS microchamber (3.7 mm in diameter) was placed over the interdigitated electrodes. Twenty microliters of the reference solution was added to the chamber, and the chamber was sealed with a coverslip to minimize evaporation. Impedance measurements were performed using an ISX-3 impedance analyzer (Sciospec Scientific Instruments GmbH, Germany) over a frequency range of 20 Hz to 30 MHz with an excitation voltage of 200 mVpp (millivolts peak-to-peak), following load compensation with a 1-kΩ resistor. The test sample was measured under identical conditions immediately after the reference. Total impedance (*Z*) and phase angle (*θ*) were calculated from the real (*Z*′) and imaginary (*Z*″) components. As the phase angle values were small, the final results were expressed as the absolute differential impedance and phase angle values between the test and reference samples, |Δ*Z*| = |*Z*_test_ − *Z*_ref_|, and |Δ*θ*| = |*θ*_test_ − *θ*_ref_| to reduce intersample variability. All downstream analyses were conducted at 100 kHz based on our previous findings [22]. Wherever possible, duplicate samples were prepared independently from the same stock to check the reproducibility of the data (see Tables S2 and S4 for SDs). Area under the curve (AUC), sensitivity, and specificity values were obtained using GraphPad Prism (v10.3.1).

#### TEM characterization

The prepared cfDNA samples were analyzed using TEM. Briefly, 10 μl of the sample was dropped on a copper-mesh-coated TEM grid and blotted from the other side of the grid. The TEM grids were then stained with UranyLess EM stain for 1 min, followed by washing with distilled water. The grids were air-dried overnight and then imaged at different magnifications using TEM (Zeiss TEM Libra 120) at 100 kV. These images were then analyzed using ImageJ software (Fiji, version 2.16) to estimate the mean of the percentage area covered by the aggregates, which was then plotted as means ± 1 SD (see Table S5 for analysis details).

### Methylation analysis of Illumina 450K and EPIC arrays from public studies

To provide biological context for the impedance signal measured by Asima Rev, we analyzed publicly available methylation array datasets from CRC tissue and blood-derived samples. We note that these datasets represent tissue gDNA or blood gDNA and therefore provide biological plausibility rather than direct characterization of the cfDNA samples used in the clinical assay. Analyses were restricted to OpenSea probes, which are most relevant to cfDNA composition given the established enrichment of cfDNA for OpenSea fragments [25].

#### Data preparation from TCGA

Illumina 450K methylation array data for colon adenocarcinoma (COAD) and rectum adenocarcinoma (READ) were downloaded from TCGA via the Genomic Data Commons (GDC) portal (https://portal.gdc.cancer.gov/). Metadata, including sample processing and clinical information, and preprocessed *β* values were obtained. The *β* values, ranging from 0 to 1, represent the mean methylation level for each of the 486,427 probes in every sample. The dataset contained 458 samples, including 413 tumor samples and 45 adjacent normal tissue controls. Each sample was annotated with age, sex, primary diagnosis, and pathologic stage using associated metadata. Probes whose values were missing in more than 20% of the samples were removed prior to downstream analysis. Principal components analysis (PCA) was performed on the top 1,000 most variable probes across all samples, and it was observed that the adjacent normal samples clustered on the basis of gender (Fig. S2). Therefore, probes on X and Y chromosomes were filtered out, along with the probes that are known to be cross-reactive [26]. In addition, the probes that fall within commonly known single-nucleotide polymorphisms (SNPs) were filtered using the dropLociWithSnps function in the Bioconductor package minfi. This function uses a list of common SNPs from dbSNP137 with a minor allele frequency of >1% within each 450K probe [27]. Filtering of probes based on missing values, low detection values, sex chromosomes, cross-reactivity, and presence in SNPs is also recommended as preprocessing steps in the majority of published research studies using methylation array data [28]. The remaining 398,783 probes were used for further analysis (Fig. S3 and Table S6).

#### Data preparation from Gene Expression Omnibus

Preprocessed *β* values were downloaded from Gene Expression Omnibus (GEO) [29] using the GEOquery package from Bioconductor [30]. In some studies, *β* values for each probe were available along with *P* values. In these cases, only probes with a *P* value of less than 0.05 in each sample were considered for analysis. Filtering was applied for probes missing in more than 20% of the samples in each study, probes on X and Y chromosomes, probes in commonly known SNPs, and probes that are known to be cross-reactive, as described in the TCGA data preparation section. In 4 studies that used 850K arrays, only probes that belong to 450K arrays were retained. The total number of probes available from source, number of probes with 450K array annotations, number of probes remaining after applying all filtering steps, and number of OpenSea probes in all studies are provided in Table S6.

#### Annotation of genomic regions

Genomic regions for each probe, such as the chromosome location and classification of each probe as belonging to a CpG island, a CpG shore, a CpG shelf, or an OpenSea region, were annotated for all studies using the IlluminaHumanMethylation450kanno.ilmn12.hg19 [31] package from Bioconductor.

#### Annotation of molecular subtypes in TCGA data

TCGA samples were annotated into 4 categories based on gene expression data—CMS1 to CMS4, 2 categories based on MSI and microsatellite stability (MSS) status, 3 categories based on CIMP-high, -low, and -negative subtypes, and v-Raf murine sarcoma viral oncogene homolog B (BRAF) mutation status based on a file released by Colorectal Cancer Subtyping Consortium [13,32].

#### Annotation of LINE-1 probes

Probes belonging to LINE-1 regions were annotated using the AnnotationHub package from Bioconductor. 450K probe coordinates (hg19) were obtained from the IlluminaHumanMethylation450kanno.ilmn12.hg19 package [33] and converted to genomic ranges. RepeatMasker annotations were downloaded and filtered to LINE-1 elements (repClass = “LINE”, repFamily = “L1”). Probe overlaps with LINE-1 intervals were identified with the findOverlaps function from the GenomicRanges package [34], and probes with at least one overlap were retained as LINE-1-associated CpGs.

#### Data quality checks

Data quality checks were performed using the methylation status of probes associated with 5 genes described by Lu *et al.* [35]. The methylation level of probes associated with NEDD4-binding protein 2 (cg13107169), EGF-like domain multiple 8 (cg16282679), chymotrypsinogen B1 (cg16863382), tetraspanin 3 (cg21377793), and zinc finger protein 690 (cg12784172) was found consistently high across 13 human tissue types and 24 cell lines. Therefore, these probes were considered similar to “housekeeping genes” in gene expression data analysis and used for quality checks on methylation array datasets.

#### Comparison of methylation between samples

Three different proxies for the mean methylation of a sample in a study were calculated—mean *β* value of all probes after filtration (*β*_mean, all_), mean *β* value of all probes annotated as OpenSea (*β*_mean, OS_), and mean *β* value of all probes annotated as LINE-1 (*β*_mean, LINE-1_). The metric used was specified in each figure and the corresponding text. For quantification of hypermethylated and hypomethylated probes in each genomic region, Δ*β* was calculated by subtracting the mean *β* value of each probe in healthy or adjacent normal samples from the mean *β* value in cancer samples. Probes with Δ*β* < −0.1 were classified as hypomethylated, and those with Δ*β* > 0.1 were classified as hypermethylated. The percentage of probes meeting these criteria in each region was reported. To assess whether our findings depended on the choice of summary statistic, we also compared diseased versus control cohorts across TCGA and GEO datasets using median *β* values as a measure of overall sample methylation and found the key hypomethylation results to be consistent (Table S7 and Note S1).

#### Statistical analyses

Statistical analyses and graphical visualizations for experimental results were performed using GraphPad Prism (v10.3.1), and methylation analyses were performed using R (v4.5.1) and Bioconductor packages, unless otherwise noted. All the schematics and graphical illustrations were prepared in Microsoft PowerPoint, unless mentioned otherwise. Unpaired Wilcoxon rank sum tests, performed using the function wilcox.test from the stats package in R, were used for group comparisons. Linear regression analyses were performed as described. All *P* values are 2-sided; *P* < 0.05 was considered statistically significant.

## Results

### Diagnostic performance of Asima Rev

The diagnostic threshold applied in this study (29 Ω) was prespecified from our prior published pan-cancer study [22], in which a threshold was established across 216 samples spanning 15 cancer types. The prior study used a chamber with slightly different dimensions from that used here. Since impedance scales linearly with the cell constant of the measurement chamber, the prior threshold was converted to the present setup using the relationship *Z*_2_ = *Z*_1_ × (*κ*_2_/*κ*_1_), where *Z*_1_ and *Z*_2_ are the thresholds in the prior and present setups, respectively, and *κ*_1_ and *κ*_2_ are the corresponding cell constants. This scaling yielded the prespecified threshold of 29 Ω applied here. This is a purely physical adjustment with no free parameters; full details of the cell constant calculation are provided in the Supplementary Materials (Note S2). No empirical optimization of this threshold was performed on the present dataset, eliminating the risk of overfitting. Of the 79 samples analyzed, only 4 appeared in the prior cohort; all remaining samples were independently collected, processed, and analyzed.

Diagnostic performance of Asima Rev was evaluated on 46 patients with CRC and 33 healthy individuals (Tables S2 and S4). Impedance spectra of test samples were recorded across a wide frequency range (20 Hz to 30 MHz) to assess the aggregation differences between healthy and cancer-derived cfDNA (Fig. 1A). cfDNA from healthy individuals consistently exhibited higher conductivity compared to cfDNA isolated from patients with cancer (Fig. 1B), while the phase angle remained low and stable across all samples (Fig. 1C). Both *Z* and Δ*Z* values plateaued between 10 kHz and 1 MHz, with the phase angles in this region remaining <2°, indicating dominantly resistive behavior. The maximum Δ*Z* was observed around 100 kHz (Fig. 1D), as previously reported by our group [22]. When Δ*Z* values were compared between healthy and CRC samples, a clear separation was observed (Fig. 1E), with stage I samples showing slightly higher mean values than more advanced stages (Fig. 1F). Age and sex were not included as covariates in this analysis, and age may contribute to some of the observed variability as discussed below. The prespecified binary classification threshold of 29 Ω yielded an AUC of 0.96 (Fig. 1H). At this threshold, the assay demonstrated an overall sensitivity of 95.65% (95% confidence interval [CI], 85.47% to 99.23%) and a specificity of 93.94% (95% CI, 80.39% to 98.92%) for CRC detection. Table S8 shows receiver operating characteristic (ROC)-derived sensitivity and specificity of |Δ*Z*| across representative diagnostic cutoffs. This includes sensitivity and specificity at representative cutoff values across the ROC curve, including the Youden-optimal cutoff (28.84 Ω) and the cutoff applied in the manuscript (29 Ω; Fig. 1E), which lies within 1% of optimal performance. Sensitivity increased with disease stage as follows: 83.33% for stage I (95% CI, 43.65% to 99.15%), 100% for stage II (95% CI, 78.47% to 100.0%), 92.3% for stage III (95% CI, 66.69% to 99.61%), and 100% for stage IV (95% CI, 77.19% to 100.0%) (Fig. 1G). The wide confidence interval for stage I sensitivity reflects the small sample size (*n* = 6) and should be interpreted with caution; these findings are considered preliminary and require confirmation in larger prospective studies. Among the patients with CRC, 6 had a history of cancer (see Table S9). Of these, 5 were correctly identified as positive by the assay, while 1 patient with stage I CRC and prior leukemia was found to be a false negative.

To assess the potential influence of preanalytical variability across sample sources, we compared differential impedance values across the 3 biobanks contributing cancer samples to this study. No statistically significant difference in |Δ*Z*| was observed across biobanks (see Fig. S1), suggesting that interbiobank preanalytical variability did not substantially confound the results.

### CRC surveillance samples analyzed using Asima Rev

**Fig. 2.**
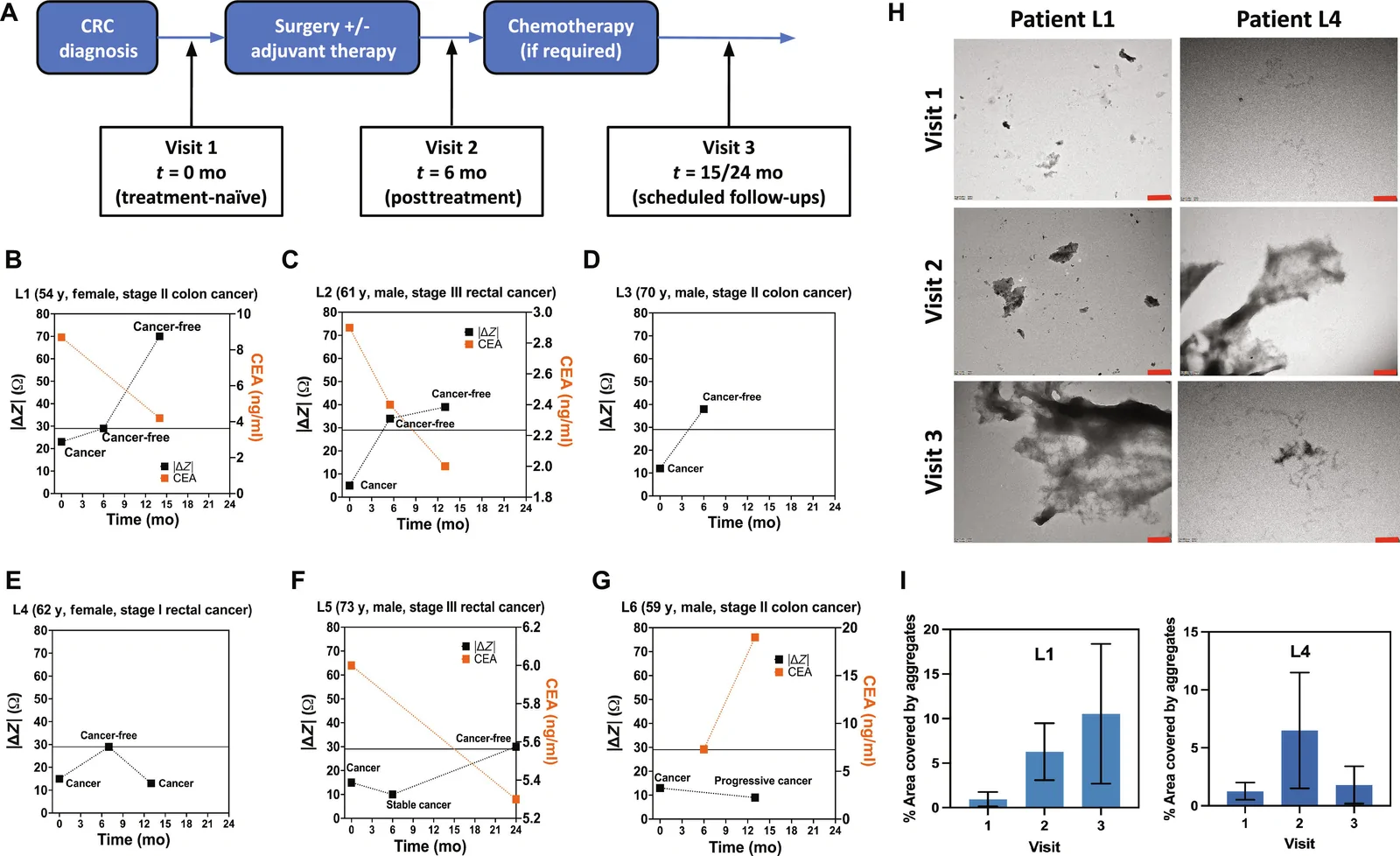
(A) Schematic showing timeline of longitudinal sample collection. (B to G) Longitudinal profiles of 6 patients with colorectal cancer (CRC) (L1 to L6) over a 24-month period. Solid black data points represent |Δ*Z*| values at 100 kHz for each time point, with *t* = 0 denoting the treatment-naïve visit at confirmed diagnosis (clinical details for this visit are provided in the title of each graph). Cancer status at each time point is based on case report forms and clinical records. The solid black horizontal line indicates the diagnostic |Δ*Z*| threshold of 29 Ω. Solid orange data points indicate blood carcinoembryonic antigen (CEA) levels, where available. (H) Representative TEM images of cfDNA samples from 3 different visits for patients L1 and L4. All the images were taken at 100 kV and 15,000× magnification. Scale bars, 500 nm. (I) TEM analysis across 90 images showing mean of area covered by the aggregates for visits 1 to 3 for the same 2 patients.

To explore the assay’s utility for CRC surveillance, we analyzed longitudinal plasma samples from 6 patients with CRC (2 females and 4 males) collected over a 24-month period, pretreatment, and posttreatment (Fig. 2A). All patients were treatment-naïve, with no neoadjuvant systemic or radiation therapy, at baseline (*t* = 0) and had stage I to stage IV CRC (Table S3). Samples were included on the basis of availability of serial collections with corresponding clinical data. Longitudinal |Δ*Z*| trajectories matched clinical status as documented in case report forms and corroborated by colonoscopy, imaging (computed tomography and magnetic resonance imaging), physical examinations, and pathology assessments. Where available, circulating carcinoembryonic antigen (CEA) levels were also included for comparison. CEA measurements were obtained from routine clinical testing performed using different assay platforms and institutional reference standards; therefore, CEA values were analyzed descriptively on an individual patient basis rather than pooled for formal statistical comparison. In most cases, increasing |Δ*Z*| values corresponded to declining CEA levels and clinical remission, whereas decreasing |Δ*Z*| aligned with disease progression (Fig. 2B to G). For example, patient L4 showed rising |Δ*Z*| after a rectal carcinoid resection at visit 1, which later declined with the emergence of a new 4-mm lung nodule seen on follow-up computed tomography, highlighting the assay’s sensitivity to emerging metastatic lesions (Fig. 2E). Patient L1 showed an increase in |Δ*Z*| after successful sigmoidectomy for removal of sigmoid COAD at visit 1. Patient L1 was given adjuvant chemotherapy until visit 2 and had no clinically adverse reports at visit 2, followed by continued positive prognosis at visit 3, with continuously declining CEA levels (Fig. 2B). In patient L5, a slight decrease in |Δ*Z*| was observed (from 15 to 10 Ω) during chemotherapy (5-fluorouracil), despite tumor shrinkage (from 5.7 mm × 0.9 mm to 2.4 mm × 0.6 mm) as confirmed by magnetic resonance imaging during visit 2. The |Δ*Z*| increased to 30 Ω, however, at visit 3 posttreatment and with no radiologic evidence of disease.

The discrepancy in |Δ*Z*| decrease in visit 2 for patient L5, despite tumor regression, may reflect lag in cfDNA clearance or other physiological influences such as biological aging, a known contributor to global hypomethylation in peripheral blood, reflecting that age-related blood cell type composition is associated with tissue-specific changes in global DNA methylation [36]. In general, our assay captured |Δ*Z*| variations despite normal age-related differences. Figure 1I shows a negative association between Δ*Z* and age in healthy individuals (slope = −0.1894, *R*^2^ = 0.0444, *P* = 0.2392), contrasted by positive slopes in patients with CRC (slope = 0.1616, *R*^2^ = 0.03204, *P* = 0.4502 and slope = 0.2690, *R*^2^ = 0.07563, *P* = 0.1739 for CRC stage I/II and CRC stage III/IV, respectively) using linear regression analysis. However, age accounted for only approximately 15% of the observed variability, suggesting that additional biological and technical factors contribute to the measured signal. Potential biological explanations include age-associated changes in cfDNA composition, cellular turnover, chronic inflammation, and epigenetic drift. Future studies involving larger age-stratified cohorts will be required to determine whether age-adjusted reference ranges are necessary for clinical implementation. Across the remaining longitudinal samples, the Asima Rev correctly reflected clinical remission or recurrence, although these patient-specific deviations highlight the need to investigate potential confounders affecting the |Δ*Z*| signal. Given the small number of longitudinal patients (*n* = 6), these findings should be interpreted as a proof-of-concept case series rather than a validated longitudinal assessment; prospective evaluation in larger cohorts is warranted.

TEM analysis of samples from all 3 longitudinal visits of patients L1 and L4 confirmed cfDNA aggregation in samples, with the extent of aggregation correlating with the |Δ*Z*| values generated by Asima Rev. At visit 1, when both patients had a confirmed diagnosis, TEM images showed minimal cfDNA aggregation (Fig. 2H), consistent with their low |Δ*Z*| values (23 and 15 Ω) below the threshold. By visit 2, when both patients were clinically cancer-free, |Δ*Z*| values increased (Fig. 2B and E), and, correspondingly, TEM revealed clear cfDNA aggregates. Patient L1 remained cancer-free at visit 3 and exhibited a high |Δ*Z*| of 70 Ω, much above the threshold, with TEM showing sustained high levels of aggregation. In contrast, patient L4 showed clinical recurrence at visit 3, accompanied by a decrease in |Δ*Z*| to 13 Ω (below the threshold) and a corresponding loss of visible cfDNA aggregation. While Fig. 2H provides representative images, all TEM fields captured in this study were quantitatively analyzed to determine average aggregate area, which aligned with previously reported methylation-dependent cfDNA structural changes (Fig. 2I). Together, these results are consistent with the proposed mechanism that cfDNA morphology and charge distribution, modulated by methylation status, may underpin the assay’s impedance-based detection signal.

### Estimating global DNA methylation levels from 450K array probes

**Fig. 3:**
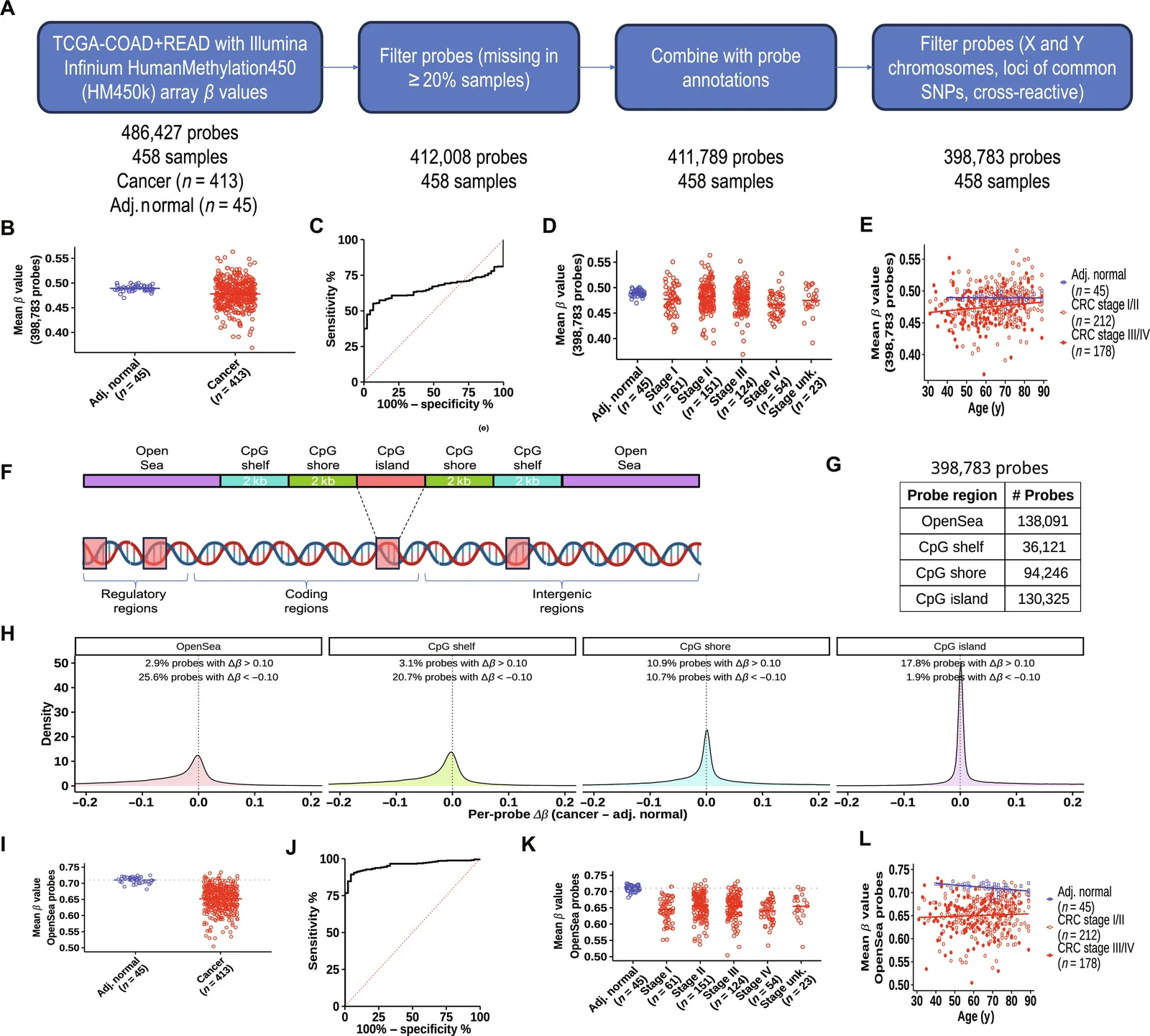
Mean methylation of OpenSea probes capturing global hypomethylation in CRC tissue. (A) An overview of methylation data preparation of The Cancer Genome Atlas (TCGA) CRC samples. (B) Comparison of mean *β* values across all 398,783 filtered probes (*β*_mean, all_) in CRC (*β*_mean, all_ = 0.4780) and adjacent normal tissue (*β*_mean, all_ = 0.4893) samples (Wilcoxon rank sum test, *W* = 6368, *P* = 0.0005). (C) ROC curve indicating low discriminatory ability AUC = 0.66; 95% confidence interval [CI], 0.61 to 0.71) for CRC detection. (D) *β*_mean, all_ for patients with CRC separated by pathologic stage. Stage unk. refers to samples whose pathologic stage is not annotated in TCGA. (E) Linear regression of *β*_mean, all_ with age in CRC samples for stage I/II (slope = 0.00043, *P* = 0.0049), stage III/IV (slope = 0.00028, *P* = 0.0566), and adjacent normal tissue (slope = −0.00001, *P* = 0.887). (F) Schematic showing higher density of CpG islands in gene regulatory regions and their surrounding genomic contexts: CpG shores (0 to 2 kb), CpG shelves (2 to 4 kb), and OpenSea regions (>4 kb). (G) Table showing number of probes belonging to each genomic region for the TCGA data. (H) Density of Δ*β* values (cancer − adjacent normal) for all probes belonging to each of the 4 genomic regions in (F). (I) Comparison of mean *β* value across all 138,091 OpenSea probes (*β*_mean, OS_) in adjacent normal tissue (*β*_mean, OS_ = 0.710) and CRC (*β*_mean, OS_ = 0.652) (Wilcoxon rank sum test, *W* = 819, *P* < 2.2 × 10^−16^). The dotted gray line shows *β*_mean, OS_ for adjacent normal tissue. (J) ROC curve indicating high discriminatory utility (AUC = 0.96; 95% CI, 0.94 to 0.98) for CRC detection. (K) *β*_mean, OS_ for patients with CRC separated by pathologic stage. (L) Linear regression of *β*_mean, OS_ with age in CRC samples for stage I/II (slope = 0.00030, *P* = 0.15), stage III/IV (slope = 0.00013, *P* = 0.53), and adjacent normal tissue (slope = −0.00034, *P* = 0.0025).

The Illumina 450K BeadChip array interrogates ~450,000 CpG loci across the genome. After excluding missing, low-quality, cross-reactive, and gender-specific probes in the combined TCGA COAD and READ datasets (Fig. 3A), mean methylation across the remaining 398,783 probes (*β*_mean, all_) was used to provide an initial estimate of global methylation differences between CRC tissue and adjacent normal tissue. Demographic and clinical characteristics of patients with CRC from TCGA are provided in Table S10. The rationale for probe filtering is provided in the Supplementary Materials (Figs. S2 and S3 and Note S3). This “global” measure indicated modest hypomethylation in CRC tissue (*β*_mean, all_ = 0.4780) compared to adjacent normal tissue (*β*_mean, all_ = 0.4893) (Fig. 3B). The difference was significant on the basis of the Wilcoxon rank sum test (*W* = 6368, *P* = 0.0005) but had a small rank biserial effect size of 0.162 that led to limited discriminative utility (ROC AUC = 0.66; 95% CI, 0.61 to 0.71) (Fig. 3C). Hypomethylation was observed across all CRC stages, with the lowest *β* values in stage IV tumors (*β*_mean, all_ = 0.4665) (Fig. 3D). To determine whether mean methylation of all probes, *β*_mean, all_, captured methylation changes with age, we performed linear regression analysis between *β*_mean, all_ and age. We found age to be a significant predictor of *β*_mean, all_ in early-stage CRC (slope = 0.00043, *P* = 0.0049 for stage I/II), but it was not a significant predictor of *β*_mean, all_ in healthy tissue (slope = −0.00001, *P* = 0.887) and late-stage CRC (slope = 0.00028, *P* = 0.0566 for stage III/IV) (Fig. 3E).

The above estimates of genome-wide methylation were constrained by the intrinsic design bias of the Illumina 450K array, which includes probes targeting CpG islands, CpG shores, CpG shelves, and OpenSea regions of the human genome (Fig. 3F) [23]. By design, the array disproportionately targets CpG islands (30.9% of all probes [37]), which constitute only ~0.7% of the genome and underrepresent OpenSea regions (36.3% of all probes [37]), which make up ~93.3% of the genome (Fig. 3G) [38]. The percentage of hypomethylated (Δ*β* < −0.1) and hypermethylated (Δ*β* > 0.1) probes across different genomic regions revealed that only 1.9% of CpG island probes were hypomethylated in CRC, compared to 25.6% of all OpenSea probes (Fig. 3H). This imbalance led *β*_mean, all_ to substantially underestimate the extent of genome-wide hypomethylation due to overrepresentation of CpG island probes. Moreover, prior studies have indicated enrichment of OpenSea fragments in cfDNA [25], further supporting their relevance for liquid biopsy assays. Accordingly, mean *β* value across OpenSea probes (*β*_mean, OS_) provided a much more accurate and clinically meaningful measure of global hypomethylation in CRC. Consistent with these findings, *β*_mean, OS_ showed a strong Pearson’s correlation [*r*(456) = 0.988, *P* < 2.2 × 10^−16^] with mean methylation of LINE-1 probes (*β*_mean, LINE-1_) (Fig. S5), which are established markers of global hypomethylation, while *β*_mean, all_ showed a substantially weaker correlation [*r*(456) = 0.829, *P* < 2.2 × 10^−16^] (Fig. S4).

Using this measure, CRC tissue samples were found to be strongly and significantly hypomethylated (*β*_mean, OS_ = 0.652) compared to adjacent normal tissue (*β*_mean, OS_ = 0.710, Wilcoxon rank sum test, *W* = 819, *P* < 2.2 × 10^−16^) (Fig. 3I) with a moderate rank biserial effect size of 0.470 that yielded a clinically meaningful ROC AUC of 0.96 and 95% CI of 0.94 to 0.98 (Fig. 3J). Although *β*_mean, OS_ showed greater variance in CRC tissue than adjacent normal tissue samples, the hypomethylation pattern was consistent across all cancer stages (Fig. 3K). Linear regression analysis of *β*_mean, OS_ with age showed significant age-related hypomethylation in adjacent normal tissue (slope = −0.00034, *P* = 0.0025) but no significant change with age in CRC tissue (slope = 0.00030, *P* = 0.15 and slope = 0.00013, *P* = 0.53 for CRC stage I/II and CRC stage III/IV, respectively) (Fig. 3L).

### Estimating DNA methylation across known molecular subtypes of CRC

**Fig. 4.**
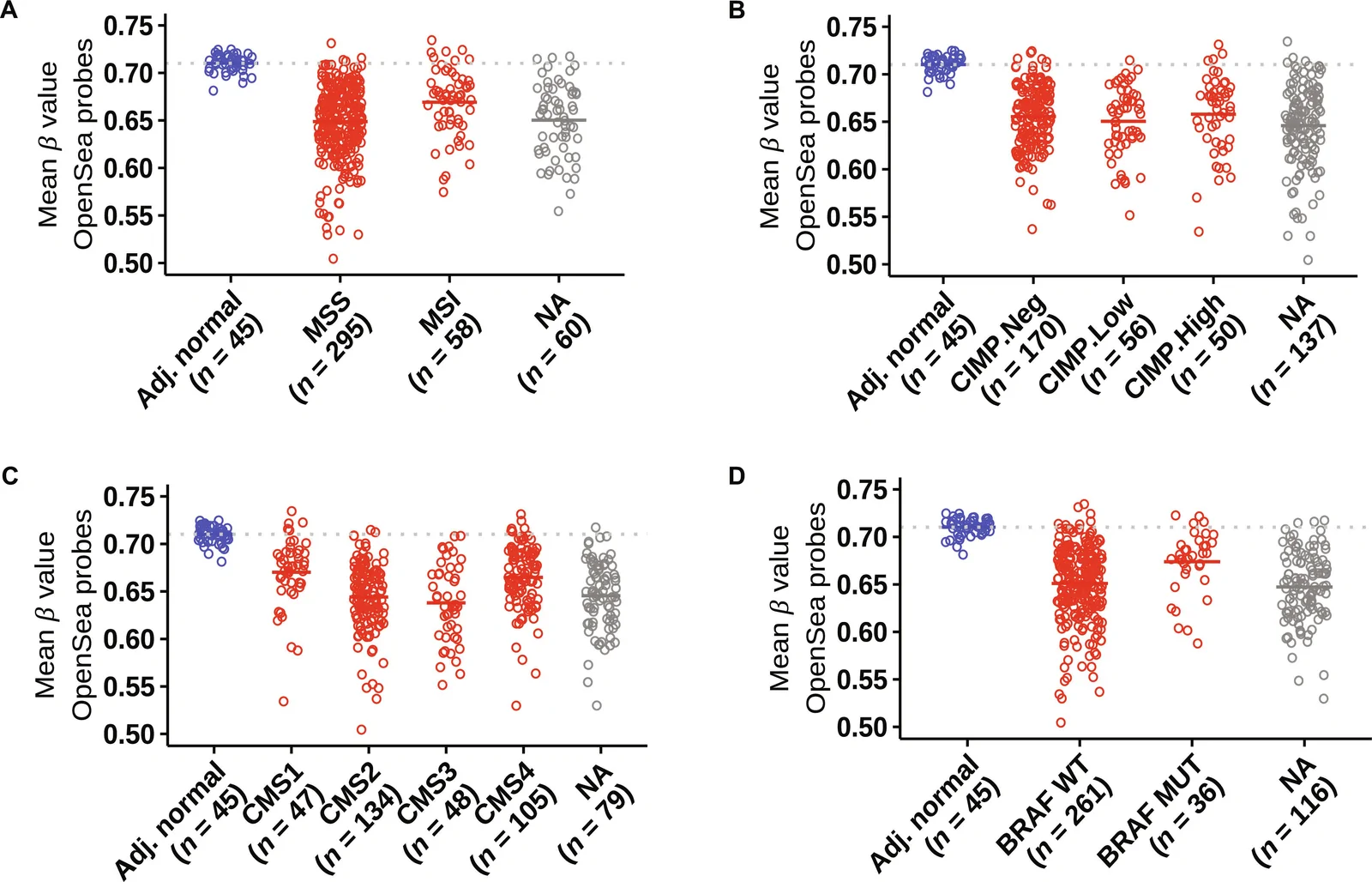
All CRC molecular subtypes showed hypomethylation compared to adjacent normal tissue in TCGA. Mean *β* value across OpenSea probes (*β*_mean, OS_) in patients with CRC with (A) microsatellite stability (MSS) (*β*_mean, OS_ = 0.649, Wilcoxon rank sum test, *W* = 347, *P* < 2.2 × 10^−16^) and microsatellite instability (MSI) (*β*_mean, OS_ = 0.669, Wilcoxon rank sum test, *W* = 325, *P* = 7.22 × 10^−11^). (B) CpG island methylator phenotype—high (CIMP.High) (*β*_mean, OS_ = 0.658, *W* = 176, *P* = 1.51 × 10^−12^), low (CIMP.Low) (*β*_mean, OS_ = 0.650, *W* = 67, *P* = 3.60 × 10^−16^), and negative (CIMP.Neg) (*β*_mean, OS_ = 0.655, *W* = 299, *P* < 2.2 × 10^−16^). (C) Consensus molecular subtypes—CMS1 (*β*_mean, OS_ = 0.670, *W* = 232, *P* = 1.13 × 10^−10^), CMS2 (*β*_mean, OS_ = 0.644, *W* = 114, *P* < 2.2 × 10^−16^), CMS3 (*β*_mean, OS_ = 0.638, *W* = 46, *P* = 1.88 × 10^−15^), and CMS4 (*β*_mean, OS_ = 0.665, *W* = 340, *P* < 2.2 × 10^−16^). (D) Wild-type v-Raf murine sarcoma viral oncogene homolog B (BRAF WT) (*β*_mean, OS_ = 0.651, *W* = 458, *P* < 2.2 × 10^−16^) and mutated BRAF (BRAF MUT) gene (*β*_mean, OS_ = 0.674, *W* = 192, *P* = 4.26 × 10^−9^) compared to adjacent normal tissue (adj. normal) (*β*_mean, OS_ = 0.710). NA refers to samples for which molecular subtype annotation was unavailable.

Next, we compared *β*_mean, OS_ across 4 independent molecular subtypes, namely, CRC with MSI and MSS; CRC with CIMP-high, CIMP-low, and CIMP-negative status; CRC with subtypes defined by Guinney *et al.* [12] based on gene expression clustering, CMS1 to CMS4; and CRC with and without BRAF mutation. Compared to adjacent normal tissue (*β*_mean, OS_ = 0.710), the mean methylation level in the MSS group was 0.649 (Wilcoxon rank sum test, *W* = 347, *P* < 2.2 × 10^−16^), and that in the MSI group was 0.669 (Wilcoxon rank sum test, *W* = 325, *P* = 7.22 × 10^−11^) (Fig. 4A). For CIMP, where differences exist in hypermethylation on CpG islands, the mean global methylation level of CIMP-high (*β*_mean, OS_ = 0.658) was similar to that of CIMP-low (*β*_mean, OS_ = 0.650) and CIMP negative (*β*_mean, OS_ = 0.655), and all were significantly lower than the adjacent normal tissue (Wilcoxon rank sum test, *W* = 176, *P* = 1.51 × 10^−12^ for CIMP high, *W* = 67, *P* = 3.60 × 10^−16^ for CIMP low, and *W* = 299, *P* < 2.2 × 10^−16^ for CIMP negative) (Fig. 4B). Across CMSs, we observed that the mean methylation across CMS1 (*β*_mean, OS_ = 0.670) and CMS4 (*β*_mean, OS_ = 0.665) was slightly higher compared to CMS2 (*β*_mean, OS_ = 0.644) and CMS3 (*β*_mean, OS_ = 0.638). Each of these subtypes were, however, significantly hypomethylated compared to healthy adjacent tissue (Wilcoxon rank sum test, *W* = 232, *P* = 1.13 × 10^−10^ for CMS1, *W* = 114, *P* < 2.2 × 10^−16^ for CMS2, *W* = 46, *P* = 1.88 × 10^−15^ for CMS3, and *W* = 340, *P* < 2.2 × 10^−16^ for CMS4) (Fig. 4C). In patients with CRC and BRAF mutation, the mean methylation level was 0.674 compared to patients with wild-type BRAF (BRAF WT) at 0.651, and both were significantly hypomethylated compared to adjacent normal tissue (Wilcoxon rank sum test, *W* = 192, *P* = 4.26 × 10^−9^ for mutated BRAF [BRAF MUT] and *W* = 458, *P* < 2.2 × 10^−16^ for BRAF WT) (Fig. 4D). Overall, we found that despite minor differences in *β*_mean, OS_ between molecular subtypes in patients with CRC, all subtypes were significantly hypomethylated compared to normal controls. These results suggest that in the absence of additional confounders, assays based on global hypomethylation would be expected to perform consistently across all CRC molecular subtypes, supporting the use of Asima Rev as a subtype-agnostic screening tool. In comparison, *β*_mean, all_ values were sensitive to hypermethylation in CpG islands and underestimated global hypomethylation across CRC molecular subtypes (Fig. S6). However, this claim could not be validated in the present study as the CMS data for biobank donors were not available. As an orthogonal validation of the subtype-specific findings, cfDNA released from CRC cell lines representing the individual CMSs could be evaluated using the Asima Rev assay and will be pursued in further studies.

### Comparing DNA methylation in healthy individuals and adjacent normal tissue from patients with CRC

**Fig. 5.**
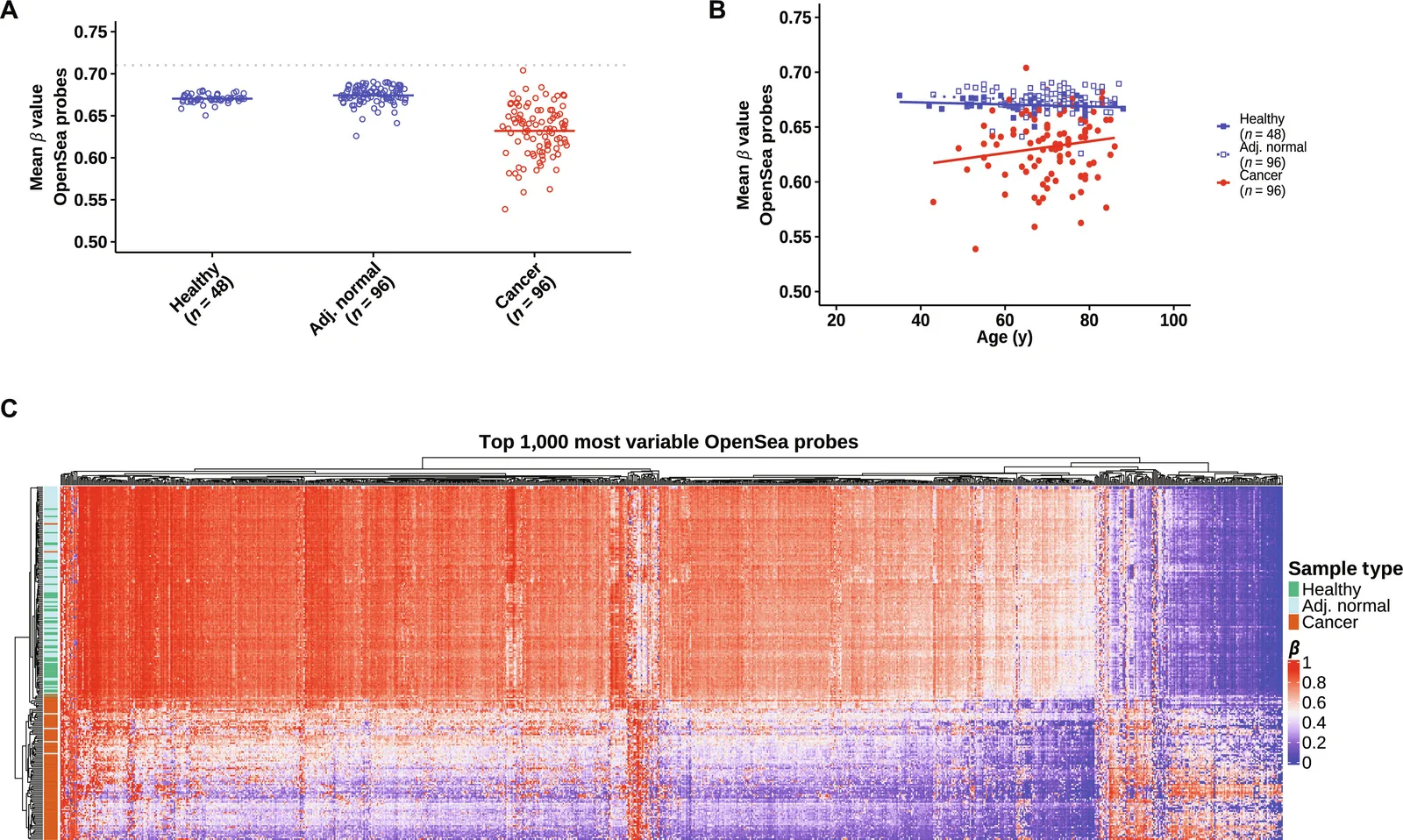
Mean methylation in healthy individuals was similar to mean methylation in adjacent normal tissue from patients with CRC in GSE131013. (A) Comparison of mean *β* values across OpenSea probes (*β*_mean, OS_) in healthy individuals (healthy) (*β*_mean, OS_ = 0.670), adjacent normal tissue (adj. normal) from patients with CRC (*β*_mean, OS_ = 0.674), and CRC tissue (cancer) (*β*_mean, OS_ = 0.632). The gray dotted line represents *β*_mean, OS_ in adjacent normal tissue from TCGA study. (B) Linear regression of *β*_mean, OS_ with age in healthy individuals (slope = −0.00009, *P* = 0.196), adjacent normal tissue (slope = −0.00013, *P* = 0.261), and CRC samples (slope = 0.0005, *P* = 0.13). (C) Clustering analysis of the top 1,000 most variable probes for healthy individuals, adjacent normal tissue from patients with CRC, and CRC tissue.

To compare DNA methylation profiles between healthy individuals and adjacent normal tissue from patients with CRC, we analyzed 4 public datasets. Although absolute *β*_mean, OS_ values for healthy individuals varied across datasets (0.670 in GSE131013 [39], 0.669 in GSE42752 [40], 0.747 in GSE48684 [41], and 0.575 in GSE199057 [42]), the methylation levels of healthy tissue from cancer-free individuals closely matched those of adjacent normal tissue from patients with CRC within each study and were consistently higher than those of CRC tissue in all except one study (Fig. 5A and Figs. S7 to S9). For example, in GSE131013, *β*_mean, OS_ was 0.670 in healthy individuals and 0.674 in adjacent normal tissue (Wilcoxon rank sum test, *W* = 3141, *P* = 3.895 × 10^−4^, with a small rank biserial effect size of 0.296), which was statistically significant but unlikely to be clinically meaningful. In contrast, *β*_mean, OS_ was 0.632 in CRC tissue compared to 0.670 in healthy individuals (Wilcoxon rank sum test, *W* = 448, *P* = 3.675 × 10^−15^, with a large rank biserial effect size of 0.655), which is statistically significant and likely to be clinically relevant (Fig. 5A).

Linear regression analysis of *β*_mean, OS_ with age indicated minimal but similar hypomethylation with age in healthy (slope = −0.00009, *P* = 0.196) and adjacent normal (slope = −0.00013, *P* = 0.261) samples and an increase in methylation with age for CRC samples (slope = 0.0005, *P* = 0.13), although the trends were not statistically significant for any group (Fig. 5B). Clustering analysis of the top 1,000 most variable OpenSea probes for all samples showed that the healthy and adjacent normal samples clustered together, indicating that these samples had similar methylation values locally at the level of individual probes and not just at a global level (Fig. 5C). These findings support the use of adjacent normal tissue in TCGA (Fig. 3) as a suitable proxy for the methylation state of healthy individuals.

### Estimating global methylation in blood gDNA of patients with CRC and autoimmune disease

**Fig. 6.**
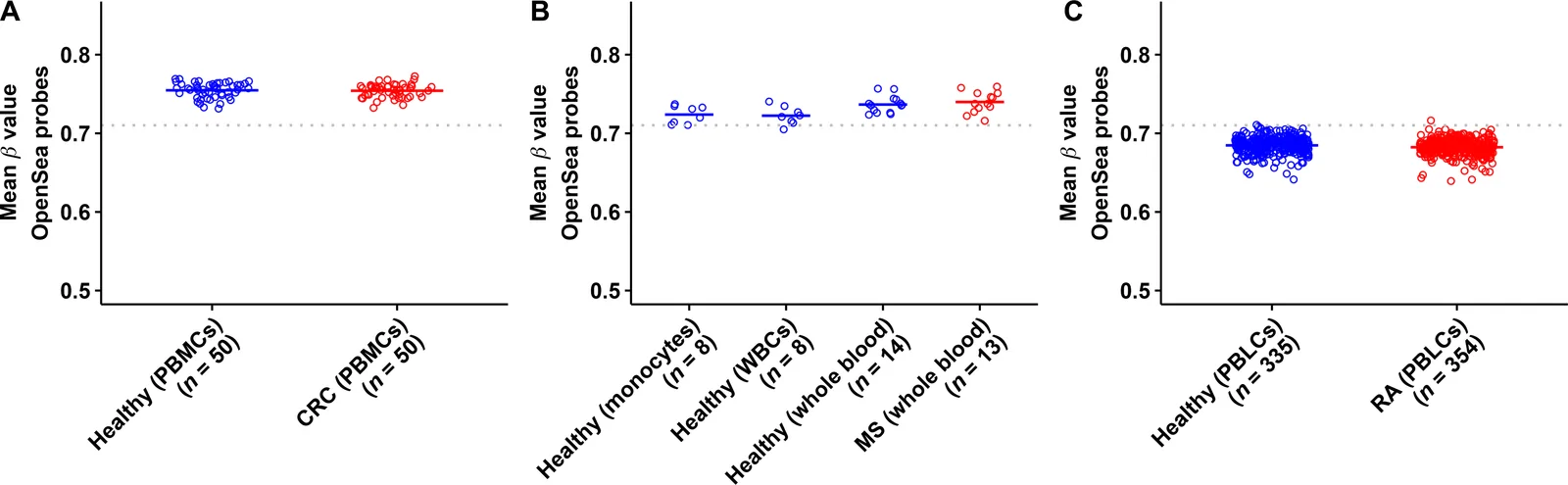
Genomic DNA (gDNA) from blood samples of patients with CRC (GSE240324) and autoimmune disease (GSE88824 and GSE42861) did not show clinically meaningful hypomethylation compared to healthy individuals. (A) Mean *β* values across OpenSea probes (*β*_mean, OS_) in gDNA from peripheral blood mononuclear cells (PBMCs) in healthy individuals (*β*_mean, OS_ = 0.755) and patients with CRC (*β*_mean, OS_ = 0.754) (*W* = 1150, *P* = 0.491). (B) *β*_mean, OS_ in whole-blood gDNA of patients with MS (*β*_mean, OS_ = 0.740) and healthy individuals (*β*_mean, OS_ = 0.737) (*W* = 106, *P* = 0.467) and gDNA from monocytes (*β*_mean, OS_ = 0.724) and white blood cells (WBCs) (*β*_mean, OS_ = 0.722) from healthy individuals. (C) *β*_mean, OS_ in gDNA from peripheral blood leucocyte cells (PBLCs) in healthy individuals (*β*_mean, OS_ = 0.685) and patients with rheumatoid arthritis (RA) (*β*_mean, OS_ = 0.682) (*W* = 50956, *P* =0.0014). The gray dotted line in each plot represents *β*_mean, OS_ in adjacent normal tissue from TCGA study.

We analyzed *β*_mean, OS_ in blood-derived gDNA from patients with CRC (GSE240324 [43]) and individuals with autoimmune diseases such as multiple sclerosis (MS) (GSE88824 [44]) and rheumatoid arthritis (RA) (GSE42861 [45]) to assess whether genomic hypomethylation in blood cells could interfere with Asima’s assay under different clinical conditions. In peripheral blood mononuclear cells (PBMCs) from patients with CRC and healthy individuals, methylation levels were nearly identical (0.755 in healthy *vs.* 0.754 in CRC; Wilcoxon rank sum test, *W* = 1150, *P* = 0.491) (Fig. 6A). Similarly, in methylation array data from patients with MS, mean methylation in whole blood was comparable between healthy individuals (*β*_mean, OS_ = 0.737) and patients with MS (*β*_mean, OS_ = 0.740; Wilcoxon rank sum test, *W* = 106, *P* = 0.467) (Fig. 6B). Monocytes and white blood cells (WBCs) from healthy individuals showed slightly lower methylation (0.724 and 0.722, respectively) than whole blood from either healthy or MS individuals. In peripheral blood leucocyte cells (PBLCs) from patients with RA, the difference between healthy (*β*_mean, OS_ = 0.685) and RA samples (*β*_mean, OS_ = 0.682) was statistically significant (Wilcoxon rank sum test, *W* = 50956, *P* = 0.0014), but likely clinically indistinguishable (Fig. 6C). Collectively, these findings suggest that the hypomethylation-associated DNA aggregation captured by Asima’s cfDNA assay is more likely to be driven by tissue-specific hypomethylation in CRC rather than by methylation changes in blood cells. Whether cfDNA methylation differs between patients with autoimmune diseases and healthy individuals remains to be determined; sequencing-based analysis of immune cells and cfDNA will be needed for definitive conclusions.

### Estimating global methylation in cfDNA samples from patients with CRC

**Fig. 7.**
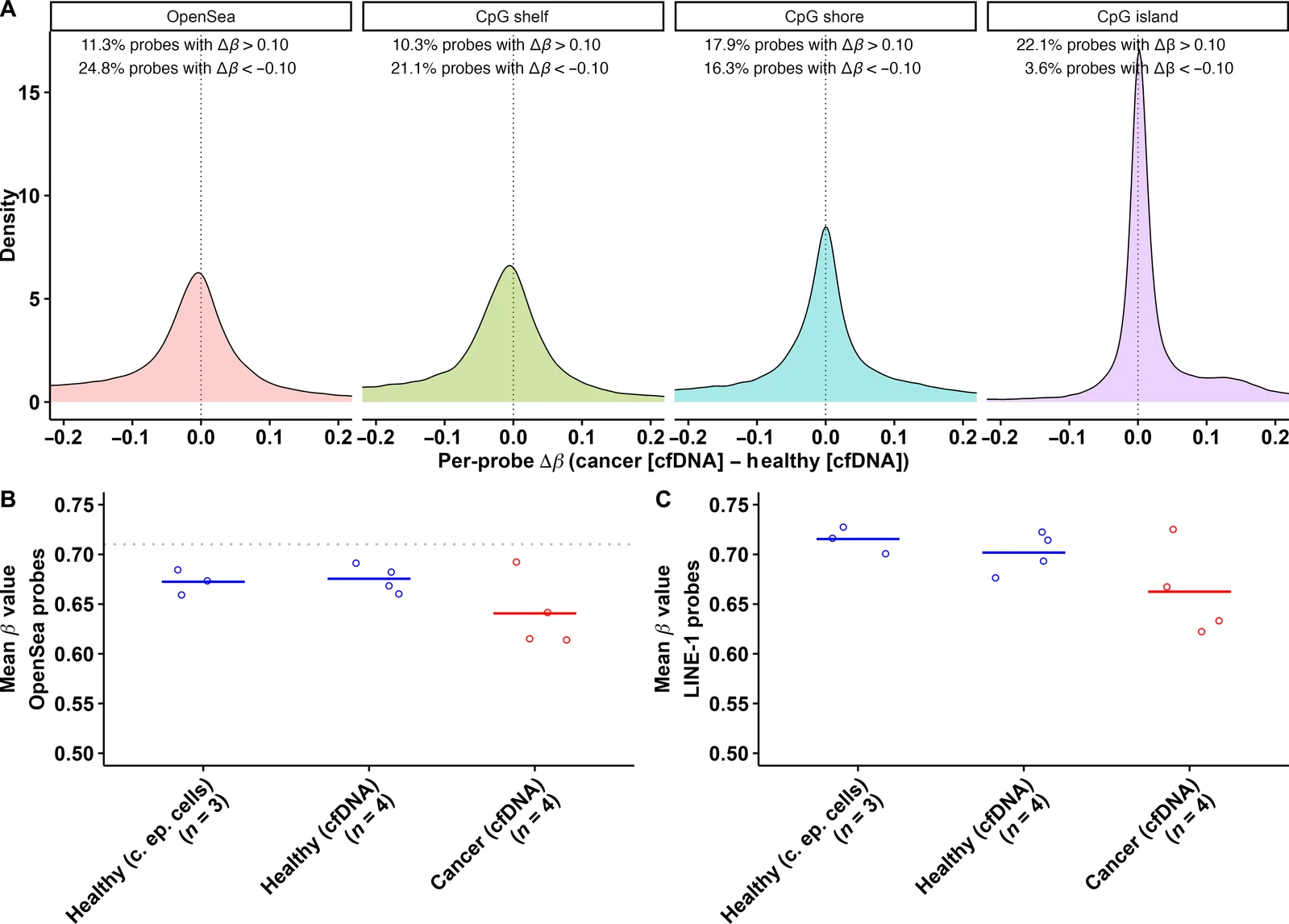
Mean methylation of OpenSea probes in cfDNA showed global hypomethylation in patients with CRC in GSE122126. (A) Density of Δ*β* values for all probes belonging to each of the 4 genomic regions on 450K array: OpenSea, CpG shelf, CpG shore, and CpG island. (B) Comparison of mean *β* value across OpenSea probes (*β*_mean, OS_) in genomic DNA (gDNA) from colon epithelial cells (c. ep. cells) (*β*_mean, OS_ = 0.672), cfDNA from healthy individuals (*β*_mean, OS_ = 0.676), and cfDNA from patients with CRC (*β*_mean, OS_ = 0.641). (C) Comparison of mean *β* value across long interspersed nucleotide element-1 (LINE-1) probes (*β*_mean, LINE-1_) in gDNA from colon epithelial cells (*β*_mean, LINE-1_ = 0.715), cfDNA from healthy individuals (*β*_mean, LINE-1_ = 0.702), and cfDNA from patients with CRC (*β*_mean, LINE-1_ = 0.662).

To establish biological plausibility for the cfDNA signal measured by Asima Rev, we examined whether global hypomethylation is detectable in cfDNA directly. Methylation array studies on cfDNA are limited because of the high DNA input requirements (~250 ng per test), which are difficult to obtain from cfDNA samples. We identified only 3 array studies using cfDNA from patients with CRC, including 2 studies (GSE110185 [20] and GSE186381 [19]) from the same research group that used pooled samples. The third cfDNA study, GSE122126 [18], did not use pooled samples but had small sample sizes with cfDNA from 4 patients with CRC and 4 healthy individuals, as well as gDNA from colon epithelial cells of 3 healthy individuals. In this study, percentages of hypomethylated (Δ*β* < −0.1) and hypermethylated (Δ*β* > 0.1) probes in CRC cfDNA compared to healthy cfDNA across different genomic regions showed 3.6% of CpG island probes to be hypomethylated compared to 24.8% of all OpenSea probes (Fig. 7A). These percentages were similar to those obtained in gDNA from TCGA samples, where 1.9% of all CpG island probes and 25.6% of all OpenSea probes were hypomethylated (Fig. 3H). The CRC cfDNA samples were also globally hypomethylated (*β*_mean, OS_ = 0.641) compared to healthy cfDNA (*β*_mean, OS_ = 0.676), as well as healthy gDNA from colon epithelial cells (*β*_mean, OS_ = 0.672) (Fig. 7B). We also examined the mean methylation levels across LINE-1 probes and found CRC cfDNA samples to be hypomethylated (*β*_mean, LINE-1_ = 0.662) compared to healthy cfDNA (*β*_mean, LINE-1_ = 0.702) and gDNA from colon epithelial cells (*β*_mean, LINE-1_ = 0.715) (Fig. 7C). The overall trend of global hypomethylation in cfDNA mirrored that observed in tissue gDNA from patients with CRC, providing biological plausibility for the Asima Rev signal. However, the sample size of this cfDNA study (*n* = 4 per group) is too small to draw statistically robust conclusions, and these findings should be interpreted as preliminary and directionally consistent with the assay mechanism rather than definitive mechanistic proof. The 2 studies with pooled cfDNA samples did not show a mean methylation difference between pooled samples from healthy individuals and patients with CRC (Figs. S10 and S11).

## Discussion

The primary finding of this study is that a diagnostic threshold established in a pan-cancer cohort of 216 samples [22] generalizes directly to an independently collected, disease-specific CRC cohort without reoptimization, yielding high sensitivity (95.65%) and specificity (93.94%). This cross-cohort generalizability supports the hypothesis that the impedance signal reflects a consistent global hypomethylation biological property of cancer cfDNA that is shared across cancer types and detectable without molecular labeling or amplification. In this study, we demonstrated that measuring cfDNA aggregation in plasma is consistent with reliably discriminating between healthy individuals and patients with CRC and that the same signal tracks with cancer recurrence in patients following curative treatment.

Our underlying hypothesis is that the electrical signal differences measured by Asima Rev are potentially driven by methylation-dependent aggregation in cfDNA. cfDNA from healthy individuals, which contains a higher number of methylated cytosines, is proposed to self-assemble into microaggregates in aqueous environments due to the hydrophobic nature of the methyl group. As methyl cytosines are progressively lost during cancer development, cfDNA may transition from microaggregated structures to a nanoaggregated state. The observed reduction in electrical signal was accompanied by reduced cfDNA aggregation in TEM images of clinical samples, consistent with the methylation-dependent aggregation mechanism established in our previous study using differentially methylated synthetic oligonucleotides [22]. Building on this observation, we analyzed available public methylation datasets to further characterize the global methylation status of gDNA from cancer and adjacent tissues and from plasma cfDNA to corroborate the potential DNA hypomethylation as a key biomarker for cancer assessment. Our analysis confirmed that global hypomethylation is consistently detectable in CRC tissue, particularly when examining OpenSea regions.

Methylation array datasets are predominantly used to study gDNA. We note that the bioinformatics analyses presented here are based on tissue gDNA and a small number of cfDNA datasets and therefore provide biological plausibility and contextual support rather than direct mechanistic characterization of the cfDNA samples assayed by Asima Rev. We acknowledge that orthogonal validation using enzymatic digestion (e.g., deoxyribonuclease I and Proteinase K treatment) would provide additional mechanistic confirmation and represents an important direction for future studies. With this caveat clearly stated, prior studies indicate that cfDNA is enriched for OpenSea fragments [25]—regions underrepresented on methylation arrays, which are strongly biased toward CpG islands (~0.7% of the genome) and gene promoters [23,24]. To address this mismatch, we restricted array analyses to OpenSea probes, aligning coverage more closely with cfDNA composition. This approach is consistent with studies using repetitive elements such as LINE-1 as global methylation proxies [46]. After truncation of the probe dataset, we observed strong concordance between Asima Rev results in cfDNA and global hypomethylation in tissue samples (specificity of 95.56% [95% CI, 88.89 to 100] and sensitivity of 89.35% [95% CI, 86.2 to 92.3] for TCGA cohort), supporting the conclusion that Asima Rev’s signal is consistent with, rather than itself proving, genome-wide methylation loss. Notably, Asima Rev’s performance exceeded that of conventional locus-specific assays, presumably because it captures structural consequences of truly global, rather than site-restricted, hypomethylation.

**Fig. 8.**
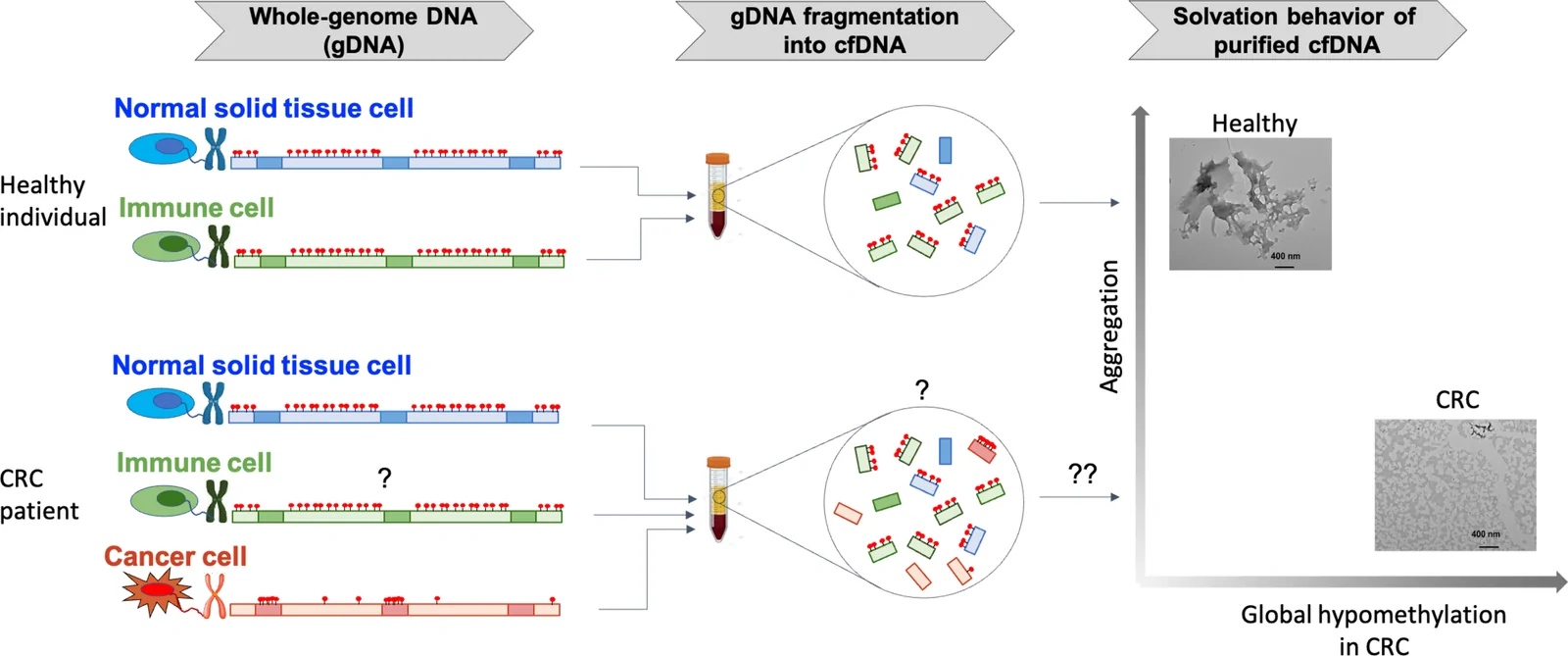
Schematic overview of the origin, proposed fragmentation and observed solvation behavior of cell-free DNA (cfDNA) in healthy individuals and patients with colorectal cancer (CRC).

A central question raised by our work is why the robust hypomethylation, so consistently observed in CRC tissue, yields inconsistent findings across the limited cfDNA methylation literature. Our analysis identified significant global hypomethylation in only one published cfDNA methylation array study but not in others. Several technical and biological factors likely contribute to this heterogeneity. Methylation arrays require relatively large DNA input (~250 ng), often necessitating investigators to pool cfDNA samples, potentially introducing bias or diluting tumor-derived signals. The cfDNA studies we analyzed varied in sample preparation protocols, pooling strategies, and cohort characteristics, all of which may influence detection sensitivity. Moss *et al.* [18] reported observable hypomethylation in individual cfDNA samples from patients with CRC, whereas Gallardo-Gomez *et al.* [19,20] used pooled samples where global hypomethylation signal may have been diluted or lost. Tumor fraction in cfDNA is known to be highly variable, with circulating tumor DNA estimated in the range of 0.1 to >10% of the total cfDNA [47]. Array-based methods, which measure average methylation across bulk cfDNA, may lack the sensitivity to detect subtle global shifts when tumor-derived DNA is sparse. Moreover, biological heterogeneity in cfDNA composition, reflecting contributions from tumor, stroma, immune cells, and normal tissue turnover [48,49], adds complexity. Tumor-derived cfDNA in patients with CRC exhibits characteristic fragmentation patterns [50], chromatin accessibility signatures [51], and nucleosome positioning [31], all of which may modulate aggregation behavior independently of or synergistically with methylation status. Our observation that immune cell gDNA does not exhibit hypomethylation in patients with CRC or autoimmune conditions suggests that changes in immune-derived cfDNA are unlikely to confound the Asima Rev signal, although sequencing-based analysis of immune cells and cfDNA will be needed for definitive answers (Fig. 8).

While we have not definitively established global hypomethylation in cfDNA of patients with CRC in this study, we have established different aggregation behavior in healthy and cancer cfDNA. Since Asima Rev measures aggregation behavior, which is a collective biophysical property, the assay may amplify even small shifts in methylation status through cooperative interactions among cfDNA molecules. This phenomenon parallels observations in DNA condensation and chromatin structure, where small changes in charge density can produce disproportionately large effects on higher-order organization. It is also possible that Asima Rev integrates multiple features beyond methylation alone. cfDNA fragment length, end motifs, and nucleosome occupancy are all altered in cancer [21] and may contribute to aggregation dynamics and provide robustness against tumor heterogeneity.

The successful transfer of the diagnostic threshold between experimental setups via cell constant normalization, without empirical reoptimization on the present dataset, has practical implications beyond this study. In clinical deployment across multiple sites, minor differences in measurement hardware would similarly be addressable through cell constant normalization rather than site-specific recalibration. This physically principled approach to threshold transfer supports the scalability and standardizability of Asima Rev as a platform technology.

In our prior study, we showed that the Asima Rev could distinguish cancer from normal across more than 15 cancer types; however, only 4 CRC samples were included in that cohort. The present study builds on those findings by demonstrating that the assay performs robustly for CRC detection specifically, with the prespecified threshold generalizing from a pan-cancer context to a disease-specific validation cohort. For context, the best-in-class Food and Drug Administration-approved blood-based cfDNA assay, Guardant Shield, reports 83% sensitivity and 90% specificity. Unlike tissue-specific assays, however, our test is pan-cancer in nature, as well as in a screening setting, would require a follow-up diagnostic test to establish tissue of origin.

Several limitations merit consideration. First, while we demonstrate strong correlation between Asima Rev signals and tissue-based global hypomethylation, direct bisulfite sequencing of the cfDNA samples assayed in this study was not performed. The mechanistic interpretation therefore relies on indirect evidence from controlled oligonucleotide experiments reported in our prior publication [22], on the cfDNA methylation array data from GSE122126 (*n* = 4), and on the established literature on CRC-associated global hypomethylation. Second, the stage I sample size (*n* = 6; 95% CI for sensitivity, 43.65% to 99.15%) is insufficient to draw firm conclusions about early-stage detection performance in CRC; this must be confirmed in larger prospective studies. Pan-cancer stage I sensitivity across this and prior work is 87% (*n* = 23). The observed detection performance in stage I patients with CRC suggests that the assay is capable of detecting low circulating tumor burdens under clinically relevant conditions but warrants more testing. A limitation of the present study is that the minimum tumor-derived cfDNA fraction required to alter the Asima Rev signal was not determined experimentally. Such analyses would require controlled dilution studies using sufficient quantities of matched healthy and tumor-derived cfDNA. Given the limited plasma volumes available from retrospective biobank samples, these experiments were beyond the scope of the current work and will be addressed in future studies. Third, the longitudinal analysis comprises only 6 patients and should be regarded as a proof-of-concept case series. Fourth, while no preprocessing variability was detected across the 3 biobanks contributing samples to this study, preanalytical variability cannot be entirely excluded as a confounding factor especially as healthy samples come from a different source altogether. Fifth, the bioinformatics analysis relies primarily on tissue gDNA methylation datasets, which provide biological context but do not directly characterize the methylation status of the cfDNA samples analyzed. Sixth, confounding contributions from nontumor cfDNA sources require further evaluation; while immune cell gDNA analysis suggests minimal methylation changes in CRC or autoimmune contexts, other inflammatory conditions, infections, and tissue injury were not considered. Finally, while we analyzed tissue gDNA across all CRC molecular subtypes, cfDNA samples stratified by subtype were not available for this study and will be evaluated in future work.

## Conclusion

In this study, we demonstrated that Asima Rev, a rapid, label-free electrical impedance assay that measures cfDNA aggregation, achieves clinically meaningful performance for CRC detection. Using a diagnostic threshold prespecified from an independent pan-cancer cohort and transferred to the present experimental setup via a physically principled cell constant normalization, the assay distinguished patients with CRC from healthy individuals with 95.65% sensitivity and 93.94% specificity across all cancer stages. The near-complete independence of the 2 cohorts (4 of 79 samples overlapping) supports the conclusion that this performance reflects genuine generalizability rather than dataset-specific optimization. In preliminary analyses, the assay also showed promise for longitudinal monitoring; however, this requires validation in larger sample sizes and ideally in a prospective study. These findings are consistent with Asima Rev as a clinically viable approach for CRC detection by leveraging cfDNA aggregation patterns that are consistent with cancer-associated global hypomethylation, as previously characterized for the Asima Rev platform [22].

While the precise molecular composition and methylation landscape of cfDNA remain active areas of investigation, our results provide compelling evidence that global hypomethylation is likely detectable in cfDNA and diagnostically actionable. Our assay supports a new paradigm in cfDNA-based cancer diagnostics, one that emphasizes structural profiling over base-by-base sequencing. Our findings from public methylation data provide biological context, bridging tissue-based observations of global hypomethylation and their translation to liquid biopsy applications for CRC, offering biological insight and generating new hypotheses to explore cancer epigenetics in cfDNA. As healthcare systems face mounting demands for accessible, cost-effective cancer screening, technologies that are simple, rapid, and accurate will be essential. The performance and operational simplicity of Asima Rev position it as a promising tool for early CRC detection and surveillance, with strong potential to increase screening uptake and expand access in underserved and resource-limited settings. Ongoing and future work will focus on mechanistic dissection of the relationship between methylation-dependent DNA structural changes and impedance behavior, including controlled perturbation experiments and orthogonal biophysical measurements.

## Data Availability

All data generated and analyzed in this study are available in the main text of the paper and the Supplementary Materials. Publicly available datasets used for analysis are referenced in the manuscript. No additional restrictions apply to the data presented in this study.

## Supplementary Materials

Supplementary 1 Note S1 to S3 Figs. S1 to S11 Table S1 to S10
