# Supplementary material for: Global Hypomethylation in Cell-Free DNA Enables Noninvasive Colorectal Cancer Screening: Results from a Retrospective Validation Study
Source: Comput Struct Biotechnol J. 2026 Aug 28;35(1):0181. doi: 10.34133/csbj.0181 (PMC13522562; doi:10.34133/csbj.0181)
Supplement: Supplementary file 1 [file csbj.0181.f1.docx]

## **Global Hypomethylation in Cell-free DNA Enables Noninvasive Colorectal Cancer Screening: Results from a Retrospective Validation Study**

## Shahdeep Kaur, Shefali Lathwal, Smita Agrawal, Tina Mahmoudi and Shalini Gupta

## ***Supplementary Information***

## **Table S1.** The list of public methylation datasets analyzed in this study.

| **No.** | **Data Source** | **Data Description** | **Sample Source** | **No. of Samples** | **Platform** |
| --- | --- | --- | --- | --- | --- |
| 1 | TCGA-COAD and TCGA-READ | CRC patients | Tissue gDNA | 458 | 450k |
| 2 | GSE131013(1) | Healthy individuals and CRC patients | Tissue gDNA | 240 | 450k |
| 3 | GSE42752(2) | Healthy individuals and CRC patients | Tissue gDNA | 63 | 450k |
| 4 | GSE48684(3) | Healthy individuals and CRC patients | Tissue gDNA | 147 | 450k |
| 5 | GSE199057(4) | Healthy individuals and CRC patients | Tissue gDNA | 229 | 850k |
| 6 | GSE240324(5) | Healthy individuals and CRC patients | PBMC gDNA | 100 | 850k |
| 7 | GSE88824(6) | Healthy individuals and Multiple Sclerosis patients | Whole blood gDNA | 83 | 450k |
| 8 | GSE42861(7) | Healthy individuals and Rheumatoid Arthritis patients | PBLC gDNA | 689 | 450k |
| 9 | GSE110185(8) | Healthy individuals and CRC patients | Whole blood pooled cfDNA | 6 | 850k |
| 10 | GSE186381(9) | Healthy individuals and CRC patients | Whole blood pooled cfDNA | 28 | 850k |
| 11 | GSE122126(10) | Healthy individuals and CRC patients | Whole blood cfDNA | 8 | 450k |

**Table S2. Clinical information of CRC patient samples along with their Asima Rev signal.** Note: Information where not available is left blank. Some ages are provided as ranges because of the data provided to us in this format by Ontario Tumor Biobank, as part of their donor de-identification strategy. These patient samples come from a single collection time point and are not pooled.

| **Sample**  **ID** | **Cancer stage** | **Site of primary**  **cancer** | **Age**  **(yrs)** | **Tumor size (cm)** | **Sex** | **cfDNA stock conc. measured using NanoDrop (ng/µL)** | **cfDNA stock conc. measured using Qubit**  **(ng/µL)** | **\|ΔZ\| at 100 kHz (ohms)** | **\|Δθ\| at 100 kHz (deg)** |
| --- | --- | --- | --- | --- | --- | --- | --- | --- | --- |
| C1 | III | Colon | 65-69 | 3.5 | F | 2.2 | 0.354 | 19.71 ± 0.71 | 0.02 |
| C2 | IV | Colon | 45-49 | 5.2 | F | 2.5 | 0.434 | 25.95 ± 2.83 | 0.00 |
| C3 | IV | Colon | 65-69 | 3.2 | M | 4.2 | 0.583 | 24.84 ± 2.12 | 0.01 |
| C4 | III | Colon | 70-74 | 1.1 | M | 3.1 | 0.206 | 20.04 ± 7.07 | 0.04 |
| C5 | III | Colon | 50-59 | 2.6 | M | 2.6 | 0.196 | 17.05 ± 2.83 | 0.01 |
| C6 | III | Colon | 60-64 | 4 | F | 3.1 | 0.519 | 20.17 ± 0.71 | 0.01 |
| C7 | I | Rectum | 65-69 |  | M | 2.9 | 0.298 | 22.29 ± 0.00 | 0.00 |
| C9 | II | Colon | 60-64 | 4.5 | M | 2.2 | 0.141 | 15.15 ± 0.00 | 0.01 |
| C10 | II | Colon | 50-54 | 2.5 | F | 2.4 | 0.398 | 24.42 ± 0.00 | 0.02 |
| C11 | IV | Colon | 60-64 | 5.5 | M | 12 | 0.472 | 26.59 ± 0.71 | 0.03 |
| C12 | II | Colon | 65-69 |  | F | 2.1 | 0.24 | 17.16 | 0 |
| C13 | III | Colon | 50-54 |  | F | 2.4 | 0.672 | 18.79 | 0 |
| C14 | IV | Colon | 65-69 |  | M | 2.6 | 1.64 | 28.05 | 0.02 |
| C15 | III | Rectum | 65-69 |  | M | 2.3 | 0.568 | 37.72 | 0.02 |
| C16 | III | Colon | 50-54 |  | M | 2.9 | 1.44 | 16.57 | 0.01 |
| C17 | I | Colon | 65-69 |  | F | 2.7 | 0.688 | 17.91 | 0.01 |
| C18 | III | Rectum | 60-64 |  | M | 5.7 | 2.18 | 15.59 | 0.04 |
| C19 | II | Colon | 55-59 |  | F | 2.1 | 0.25 | 15.02 | 0 |
| C20 | I | Colon | 70-74 |  | F | 3.1 | 0.618 | 37.86 | 0.01 |
| C21 | IV | Colon | 50-54 |  | F | 2.8 | 1.48 | 10.37 | 0.01 |
| C22 | IV | Colon | 55-59 |  | M | 3.0 | 0.38 | 15.58 | 0.03 |
| C23 | II | Colon | 55-59 |  | F | 2.3 | 0.421 | 12.21 | 0.01 |
| C24 | IV | Colon | 75-79 |  | F | 3.2 | 1.18 | 13.5 | 0.01 |
| C25 | III | Colon | 60-64 |  | F | 3.3 | 1.36 | 13.7 | 0.03 |
| C26 | II | Colon | 76 |  | M | 1.3 |  | 7.78 ± 1.07 |  |
| C27 | IV | Colon | 57 |  | F | 3.6 |  | 13.39 ± 0.18 |  |
| C28 | III | Colon | 53 |  | M | 1.8 |  | 1.72 ± 0.02 |  |
| C29 | II | Colon | 72 |  | M | 5.7 |  | 18.23 ± 0.54 |  |
| C35 | IV | Colon | 55 | 5.9 | F | 36.4 | 21.5 | 12.02 | 0.05 |
| C36 | II | Rectum | 60 |  | F | 3.3 | 0.509 | 17.15 | 0.02 |
| C37 | I | Colon | 75 | 2.4 | F | 2.3 | 0.389 | 27.19 | 0.02 |
| C38 | IV | Colon | 53 |  | F | 3.9 | 0.943 | 3.89 | 0.01 |
| C39 | II | Colon | 65 | 3.5 | M | 2.2 | 0.718 | 11.97 | 0.02 |
| C40 | IV | Colon | 75 |  | M | 2.3 | 0.756 | 23.87 | 0.01 |
| C41 | IV | Colon | 70 | 5.0 | M | 2.5 | 0.626 | 9.14 | 0.01 |
| C42 | IV | Rectum | 65 |  | M | 2.7 | 0.719 | 17.54 | 0 |
| C43 | II | Colon | 71 |  | M | 4.4 | 1.55 | 22.22 | 0.04 |
| C44 | I | Rectum | 63 |  | F | 4.6 | 1.14 | 28.53 | 0.01 |
| C45 | III | Rectum | 57 |  | M | 6.9 | 2.19 | 15.84 | 0.02 |
| C46 | II | Colon | 47 |  | F | 4.5 | 1.47 | 12.6 | 0.02 |
| L1 | II | Colon | 54 |  | F | 4.9 | 0.955 | 23.2 | 0.03 |
| L2 | III | Rectum | 61 |  | M | 2.7 | 0.484 | 5.8 | 0.04 |
| L3 | II | Colon | 70 |  | M | 5.7 | 1.13 | 12.36 | 0.02 |
| L4 | I | Rectum | 62 |  | F | 2.2 | 0.611 | 15.15 | 0.01 |
| L5 | III | Rectum | 73 |  | M | 4.2 | 0.493 | 14.6 | 0.10 |
| L6 | II | Colon | 59 |  | M | 8.6 | 5.78 | 13.74 | 0.11 |

**Table S3.** Clinical information of CRC patient samples used for longitudinal data analysis. Information where not available is left blank. NED: No Evidence of Disease

| **Sample**  **ID** | **Cancer stage at diagnosis** | **Site of primary**  **cancer** | **Age at diagnosis**  **(yrs)** | **Sex** | **CEA (ng/mL)** | **Clinical Cancer status at Visit** | **cfDNA stock conc. measured using NanoDrop (ng/µL)** | **cfDNA stock conc. measured using Qubit**  **(ng/µL)** | **\|ΔZ\| at 100 kHz (ohms)** | **\|Δθ\| at 100 kHz (deg)** |
| --- | --- | --- | --- | --- | --- | --- | --- | --- | --- | --- |
| L1V1 | II | Colon | 54 | F | 8.7 | Active disease | 4.9 | 0.955 | 23.2 | 0.03 |
| L1V2 | II | Colon | 54 | F |  | NED | 4.9 | 2.72 | 28.74 | 0.02 |
| L1V3 | II | Colon | 54 | F | 4.2 | NED | 8.1 | 0.935 | 70.92 | 0.02 |
| L2V1 | III | Rectum | 61 | M | 2.9 | Active disease | 2.7 | 0.484 | 5.8 | 0.02 |
| L2V2 | III | Rectum | 61 | M | 2.4 | NED | 3.7 | 1.39 | 35.22 | 0.04 |
| L2V3 | III | Rectum | 61 | M | 2.0 | NED | 5.7 | 1.37 | 39.09 | 0.02 |
| L3V1 | II | Colon | 70 | M |  | Active disease | 5.7 | 1.13 | 12.36 | 0.06 |
| L3V2 | II | Colon | 70 | M |  | NED | 3.1 | 1.16 | 37.95 | 0.01 |
| L4V1 | I | Rectum | 62 | F |  | Active disease | 2.2 | 0.611 | 15.15 | 0.01 |
| L4V2 | I | Rectum | 62 | F |  | NED | 2.6 | 0.672 | 29.43 | 0.01 |
| L4V3 | I | Rectum | 62 | F |  | Active disease | 3.2 | 0.523 | 12.72 | 0.01 |
| L5V1 | III | Rectum | 73 | M | 6 | Active disease | 4.2 | 0.493 | 14.6 | 0.1 |
| L5V2 | III | Rectum | 73 | M |  | Active disease | 7.4 | 2.34 | 9.63 | 0.04 |
| L5V3 | III | Rectum | 73 | M | 5.3 | NED | 3.0 | 1.53 | 30.05 | 0.04 |
| L6V1 | II | Colon | 59 | M | 7.3 | Active disease | 8.6 | 5.78 | 13.74 | 0.11 |
| L6V2 | II | Colon | 59 | M | 19 | Progressive disease | 8.7 | 2.67 | 9.32 | 0 |

**Table S4.** Clinical information of healthy individual samples used for cfDNA extraction. Information where not available is left blank.

| **Sample ID** | **Age (yrs)** | **Sex** | **cfDNA stock conc. measured using NanoDrop (ng/µL)** | **cfDNA stock conc. measured using Qubit (ng/µL)** | **\|ΔZ\| at 100 kHz (ohms)** | **\|Δθ\| at 100 kHz (deg)** |
| --- | --- | --- | --- | --- | --- | --- |
| H1 | 56 | M | 4.6 | 0.144 | 52.5 ± 2.83 | 0.01 |
| H2 | 63 | F | 3.3 | 0.238 | 45.17 ± 0.71 | 0.01 |
| H3 | 68 | F | 4.8 | 0.418 | 30.36 ± 0.71 | 0.02 |
| H4 | 59 | F | 3.8 | 0.336 | 31.62 ± 1.41 | 0.01 |
| H5 | 58 | M | 3.9 | 0.809 | 34.69 ± 0.71 | 0.02 |
| H6 | 54 | F | 3.2 | 0.287 | 32.69 ± 0.22 | 0.05 |
| H7 | 45 | M | 2.9 | 0.223 | 35.99 ± 0.71 | 0.01 |
| H8 | 60 | M | 3.1 | 0.368 | 38.27 ± 0.00 | 0.02 |
| H9 | 57 | M | 3.1 | 0.479 | 46.97 ± 5.66 | 0.01 |
| H11 | 71 | M | 3.6 | 0.362 | 49.85 ± 6.36 | 0.05 |
| H12 | 55 | F | 2.6 | 0.421 | 24.82 ± 0.71 | 0.03 |
| H13 | 54 | F | 3.8 | 0.246 | 41.06 ± 1.41 | 0.08 |
| H14 | 62 | M | 3.4 | 0.481 | 37.47 ± 0.71 | 0.02 |
| H15 | 48 | F | 3.5 | 0.318 | 46.14 ± 2.83 | 0.03 |
| H16 | 60 | F | 1.5 | 0.247 | 29.15 ± 0.00 | 0.03 |
| H17 | 63 | M | 5.2 | 0.497 | 35.41 ± 2.12 | 0.03 |
| H18 | 48 | F | 4.0 | 0.217 | 47.7 ± 5.66 | 0.04 |
| H19 | 51 | F | 4.8 | 0.193 | 58.33 ± 1.41 | 0.04 |
| H20 | 50 | M | 2.9 | 0.387 | 40.53 ± 1.41 | 0.04 |
| H21 | 54 | F | 1.7 | 0.417 | 32.94 ± 1.41 | 0.01 |
| H22 | 57 | M | 2.4 | 0.435 | 43.14 ± 2.83 | 0.03 |
| H23 | 44 | F | 3.0 | 0.297 | 34.81 ± 0.71 | 0.02 |
| H24 | 35 | F | 3.9 | 0.319 | 48.71 ± 0.71 | 0.04 |
| H25 | 50 | F | 1.4 |  | 42.97 ± 0.96 |  |
| H26 | 47 | M | 1.2 |  | 35.76 ± 1.39 |  |
| H27 | 77 | M | 2.1 |  | 34.44 ± 1.71 |  |
| H28 | 55 | F | 3.1 |  | 40.36 ± 0.60 |  |
| H29 | 75 | F | 2.8 |  | 34.51 ± 0.94 |  |
| H30 | 62 | F | 4.3 |  | 36.82 ± 0.94 |  |
| H31 | 66 | F | 3.3 |  | 33.53 ± 0.74 |  |
| H32 | 68 | M | 2.3 |  | 37.18 ± 1.29 |  |
| H33 | 57 | M | 2.6 |  | 41.08 ± 0.68 |  |
| H34 | 53 | M | 5.0 |  | 18.09 ± 0.74 |  |

## **Table S5**. Process of analysis of TEM images using ImageJ to calculate cfDNA aggregates in CRC surveillance.

| (1) Open TEM Image in Fiji (ImageJ). | (2) Subtract image background to make image more uniform given varying background fluorescence. | (3) Adjust image threshold to create a binary mask (all values set to either 0 or 1, black or white). |
| --- | --- | --- |
|  |  |  |
| (3) Erode then dilate the binary mask to remove the salt and pepper noise, then enhance the large pixel aggregates. | (4) Get an image histogram to derive the number of pixels at each binary value (0 and 1, black or white). | (5) Derive percentage of black pixels relative to all pixels, representing the percent area of the total image covered by aggregates. |
|  |  | No. white pixels: 769803; No. black pixels: 16629; No. total pixels: 786432; **Percentage black pixels or aggregates: 2.11** |

**Table S6.** Number of probes available and used for analysis in each dataset. A total of 125111 OpenSea probes were common across all datasets.

| **No.** | **Data Source** | **Total # of probes available** | **# of probes with 450k annotations** | **# of probes after filtering** | **# of probes annotated as OpenSea** |
| --- | --- | --- | --- | --- | --- |
| 1 | TCGA-COAD and TCGA-READ | 486427 | 485512 | 398783 | 138091 |
| 2 | GSE131013(1) | 485577 | 485512 | 429944 | 152026 |
| 3 | GSE42752(2) | 485577 | 485512 | 429943 | 152026 |
| 4 | GSE48684(3) | 485577 | 485512 | 429944 | 152026 |
| 5 | GSE199057(4) | 865918 | 452453 | 400942 | 142532 |
| 6 | GSE240324(5) | 728310 | 369467 | 365981 | 130500 |
| 7 | GSE88824(6) | 485512 | 485512 | 429944 | 152026 |
| 8 | GSE42861(7) | 485577 | 485512 | 429944 | 152026 |
| 9 | GSE110185(8) | 866895 | 453093 | 401400 | 142664 |
| 10 | GSE186381(9) | 865859 | 452453 | 400962 | 142534 |
| 11 | GSE122126(10) | 423213 | 423213 | 398218 | 139885 |

**Table S7.** Sensitivity of global methylation comparison between disease and control samples to the choice of summary statistic (mean versus median). For each cohort (disease and control), every sample's global methylation was summarized in two ways, as mean β-value across the probe set (β*_mean, ProbeSet_*) and as median β-value across the same probe set (β*_median, ProbeSet_*). The rows in bold represent comparisons with significant hypomethylation in diseased samples compared to controls using both mean and median. The rows in regular font represent comparisons with no significant methylation difference in diseased samples compared to controls using both mean and median. The row in italics represents a comparison where the difference was significant using mean, but not significant using median.

| **Dataset** | **Contrast (disease vs. control)^1^** | **N (case/**  **control)^2^** | **Probe**  **set^3^** | **Rank corr. (Spearman)^4^** | **Δβ (β*_mean, ProbeSet_*)^5^** | **p-val. (β*_mean, ProbeSet_*)^6^** | **Δβ (β*_median, ProbeSet_*)^7^** | **p-val. (β*_median, ProbeSet_*)^8^** |
| --- | --- | --- | --- | --- | --- | --- | --- | --- |
| **TCGA** | **CRC vs. Adj. Normal** | **413/45** | **All probes** | **0.98** | **-0.011** | **0.001** | **-0.036** | **4.69e-05** |
| **TCGA** | **CRC vs. Adj. Normal** | **413/45** | **OpenSea** | **0.86** | **-0.058** | **9.24e-24** | **-0.065** | **1.99e-15** |
| **GSE131013** | **CRC vs. Adj. Normal** | **96/96** | **OpenSea** | **0.85** | **-0.042** | **6.82e-24** | **-0.044** | **1.78e-12** |
| **GSE42752** | **CRC vs. Adj. Normal** | **22/22** | **OpenSea** | **0.87** | **-0.035** | **1.96e-07** | **-0.036** | **2.18e-04** |
| **GSE48684** | **CRC vs. Adj. Normal** | **64/24** | **OpenSea** | **0.81** | **-0.034** | **4.76e-07** | **-0.039** | **6.68e-07** |
| GSE199057 | CRC vs. Adj. Normal | 77/80 | OpenSea | 0.89 | -0.002 | 0.843 | -0.001 | 0.431 |
| GSE240324 | CRC vs. Healthy | 50/50 | OpenSea | 0.97 | -0.001 | 0.493 | 0.001 | 0.855 |
| GSE88824 | Multiple Sclerosis versus Healthy | 13/14 | OpenSea | 0.93 | 0.003 | 0.488 | 0.001 | 0.650 |
| *GSE42861* | *Rheumatoid Arthritis vs. Healthy* | *354/335* | *OpenSea* | *0.93* | *-0.002* | *0.001* | *-0.001* | *0.251* |
| GSE110185 | CRC vs. Healthy | 2/2 | OpenSea | 1.00 | -0.024 | sample size too small | -0.030 | sample size too small |
| GSE186381 | CRC vs. Healthy | 15/13 | OpenSea | 0.97 | -0.003 | 0.586 | -0.006 | 0.555 |
| GSE122126 | CRC vs. Healthy | 4/4 | OpenSea | 0.98 | -0.035 | sample size too small | -0.038 | sample size too small |

1. Cohorts being compared within the dataset
2. Number of samples in the disease and control groups for the dataset
3. Set of probes used in the global methylation calculation
4. Spearman rank correlation between β*_mean, ProbeSet_* and β*_median, ProbeSet_* across all samples in the dataset, quantifying whether the two statistics order samples consistently. Values close to 1 indicate the same ordering of samples
5. Difference in mean β*_mean, ProbeSet_* between diseased and control samples. A negative value indicates hypomethylation in the diseased cohort
6. p-value calculated for unpaired Wilcoxon rank-sum test between diseased and control samples using β*_mean, ProbeSet_* as the measure of global methylation of each sample
7. Difference in mean β*_median, ProbeSet_* between diseased and control samples. A negative value indicates hypomethylation in the diseased cohort
8. p-value calculated for unpaired Wilcoxon rank-sum test between diseased and control samples using β*_median, ProbeSet_* as the measure of global methylation of each sample

**Note S1. Assessment of the choice of summary statistic, mean or median β-value across probes on global hypomethylation analysis of diseased samples compared to controls.**

Global methylation for each sample measured using methylation arrays has been summarized as the mean β-value of a set of probes (β*_mean, all_* and β*_mean, OS_*). Since β-values are bounded and non-normally distributed, we assessed whether our cohort comparisons were sensitive to this choice by repeating diseased versus control comparisons with the median β-value of a set of probes as the measure of global methylation of a sample. For both mean and median-based calculations, we compared Spearman rank correlation to quantify whether the two statistics order samples consistently. We calculated the methylation difference between diseased and control samples in each dataset as the difference in mean global methylation of diseased cohort and mean global methylation of control cohort. We also compared whether the difference in global methylation of diseased cohort and control cohort for each dataset was statistically significant using unpaired Wilcoxon rank-sum test and compared the p-values for this test using both mean and median-based global methylation (Table S7).

Across all datasets, mean and median based global methylation calculations were strongly correlated (Spearman ρ = 0.81 - 1.00), indicating that the two statistics order samples consistently. Across all but one dataset, where global hypomethylation in diseased cohort was significant (p-value < 0.05) compared to control when using mean, global hypomethylation was also significant when using median and the difference in mean global methylation of cohorts was larger when using median of OpenSea probes for calculating global methylation of a sample compared to using mean (TCGA Δβ = -0.058 [mean] vs -0.065 [median], GSE131013 Δβ = -0.042[mean] vs -0.044[median]; GSE42752 Δβ = -0.035[mean] vs -0.036[median]; GSE48684 Δβ = -0.034[mean] vs -0.039[median]). In GSE42861, we observed significant global hypomethylation in rheumatoid arthritis (RA) samples compared to controls when using mean (p-value = 0.001) but not using median (p-value = 0.25). However, the difference in β-values between RA samples and controls is an order of magnitude smaller than in datasets showing clear hypomethylation (Δβ_mean_ = -0.002) and not likely to be clinically meaningful. Using median indicates no significant difference between RA and control samples, which is also the conclusion we reached in the manuscript. For the remaining datasets, there was no significant methylation difference between diseased and control cohorts using either mean or median. Therefore, using median β-values as a measure of global methylation of a sample instead of mean β-values does not alter any conclusion in our study.

**Table S8. ROC-derived sensitivity and specificity of |ΔZ| across representative diagnostic cutoffs.** Sensitivity and specificity at representative cutoff values across the ROC curve, including the Youden-optimal cutoff (28.84 Ω) and the cutoff applied in the manuscript (29 Ω, Fig. 1e), which lies within 1% of optimal performance. The ROC performance from healthy (n = 33) and cancer (n = 46) samples yielded an AUC of 0.96, indicating strong discriminative performance.

| **\|ΔZ\| Cutoff (Ω)** | **Sensitivity (%)** | **Specificity (%)** | **Youden Index (J)** | **Remarks** |
| --- | --- | --- | --- | --- |
| 17.10 | 52.17 | 100.00 | 52.17 | — |
| 20.11 | 69.57 | 96.97 | 66.54 | — |
| 24.15 | 80.43 | 96.97 | 77.40 | — |
| 26.89 | 89.13 | 93.94 | 83.07 | — |
| 28.29 | 93.48 | 93.94 | 87.42 | — |
| 28.84 | 95.65 | 93.94 | 89.59 | Youden-optimal cutoff |
| **29 (used)** | **95.65** | **93.94** | **89.59** | **Cutoff used in manuscript (Fig. 1e)** |
| 32.16 | 95.65 | 84.85 | 80.50 | — |
| 34.48 | 95.65 | 72.73 | 68.38 | — |
| 37.00 | 95.65 | 51.52 | 47.17 | — |
| 40.45 | 100.00 | 39.39 | 39.39 | — |
| 43.06 | 100.00 | 27.27 | 27.27 | — |

AUC = 0.96 (calculated by trapezoidal integration of the full ROC curve). Youden Index (J) = Sensitivity + Specificity − 100; the maximum J (89.59) occurs at 28.84 Ω, closely matching the 29 Ω cutoff used for classification in the main text.

## **Table S9.** Samples with history of cancer.

| Patient clinical status | No. of patients with history of cancer | Past cancer type | Asima Rev outcome |
| --- | --- | --- | --- |
| Healthy | 0 | N/A | - |
| CRC stage I | C20 | Leukemia | FN |
| CRC stage II | 0 | N/A | - |
| CRC stage III | C1 | Skin | TP |
| CRC stage IV | C3, C14, C38, C41 | Lung (C3),  CRC (C14, C38, C41) | TP |

## **Table S10.** Demographic and clinical characteristics of CRC patients in TCGA.

**Note S2. Cell constant calculations.** The diagnostic threshold established in our prior study^20^ was determined using a 3.5 mm diameter PDMS chamber whereas a 3.7 mm diameter PDMS chamber was used in the present study. In both cases, the PDMS was punched using a 4 mm biopsy punch, however, given the nature of the punch and the actual stiffness of the PDMS slab used, the final size of the hole created varied slightly across the two studies. The size of the hole (which defines the exposed sensor area) has direct bearing on the system’s cell constant.

The cell constant (𝛋) of any system is determined by measuring the impedance (Z) of known conductivity standards and plotting these values against the true values of resistivity (⍴) as shown in the figure below. The slope (𝛋 = Z/⍴) of 27.2 m^-1^ in this plot is the measured cell constant. In our case, Z is reported at 100 kHz as it’s considered equivalent to resistance at the lowest phase angle across the frequency spectra.

Since impedance scales linearly with the cell constant of the measurement chamber, the prior threshold was converted to the present setup using the relationship: Z₂ = Z₁ × (κ₂/κ₁), where Z₁ and Z₂ are the thresholds in the prior and present setups, respectively, and κ₁ and κ₂ are the corresponding cell constants. The ratio of κ₂/κ₁ was found to be 0.85 from the respective surface areas of the two chambers. This scaling yielded the pre-specified threshold of 29 Ω applied in the present study. This is a purely physical adjustment with no free parameters. As an internal consistency check, the impedance of the HEPES reference solution was confirmed to scale by the same factor between setups, validating the cell constant relationship.

**Fig. S1. A scatter plot showing |ΔZ| values at 100 kHz in healthy and CRC patients across biobank sources.** Horizontal lines indicate the mean |ΔZ| for each group. The dotted line indicates the diagnostic threshold of 29 Ω. No statistically significant difference in |ΔZ| was observed across biobanks for cancer samples supporting the absence of systematic pre-analytical processing variability.

## **Fig. S2.** Principal Components Analysis (PCA) plot of top 1000 most variable probes from 486,427 total probes in TCGA without any filtering, colored by **(a)** cancer status and **(b)** gender.

**Fig. S3.** PCA plot of top 1000 most variable probes from 398,783 total probes in TCGA after filtering, colored by **(a)** cancer status and **(b)** gender.

**Note S3. A discussion on why filtering of probes is required for Illumina Infinium methylation arrays.** Illumina Infinium methylation arrays use 50bp probes to query methylation status at various sites on the genome at a single base resolution. Before performing analysis on these data, it is common practice to filter out probes whose detection p-values are greater than a threshold, probes that occur in commonly known regions with single nucleotide polymorphisms (SNPs) that may affect CpG sites and probes that have shown to be cross-reactive. In addition, the PCA plots without removing probes on X and Y chromosomes showed that adjacent normal tissue samples cluster based on gender (Fig. S2), indicating that gender is a significant source of variation. Removing the probes from X and Y chromosomes led to cancer status being the main source of variance in the data (Fig. S3), enabling us to draw conclusions applicable to all samples.

**Fig. S4.** Correlation between mean β-value of LINE-1 probes and mean β-value of OpenSea probes in TCGA.

## **Fig. S5.** Correlation between mean β-value of all probes after filtering and mean β-value of LINE-1 probes in TCGA.

**Fig. S6.** Mean β-value for all probes (β*_mean, all_*) in CRC patients with **(a)** microsatellite stability (MSS) and microsatellite instability (MSI); **(b)** CpG Island Methylator Phenotypes - High (CIMP.High), Low (CIMP.Low) and Negative (CIMP.Neg); **(c)** Consensus Molecular Subtypes (CMS) 1-4, and; **(d)** wild type (BRAF WT) and mutated (BRAF MUT) BRAF gene compared to adjacent normal tissue (Adj. Normal). NA refers to samples for which molecular subtype annotation was unavailable.

**Fig. S7.** Comparison of mean β-values across OpenSea probes (β*_mean, OS_*) in healthy individuals (Healthy), adjacent normal tissue (Adj. Normal) from CRC patients, and CRC tissue (Cancer) in GSE42752. The gray line represents β*_mean, OS_* in adjacent normal tissue from the TCGA study.

**Fig. S8.** Comparison of mean β-values across OpenSea probes (β*_mean, OS_*) in healthy individuals (Healthy), adjacent normal tissue (Adj. Normal) from CRC patients, Adenoma patients (Adenoma) and CRC tissue (Cancer) in GSE48684. The gray line represents β*_mean, OS_* in adjacent normal tissue from the TCGA study.

**Fig. S9.** Comparison of mean β-values across OpenSea probes (β*_mean, OS_*) in healthy individuals (Healthy), adjacent normal tissue (Adj. Normal) from CRC patients, and CRC tissue (Cancer) in GSE199057. The gray line represents β*_mean, OS_* in adjacent normal tissue from the TCGA study.

**Fig. S10.** Comparison of **(a)** mean β-value across OpenSea probes (β*_mean, OS_*) and **(b)** mean β-value across LINE-1 probes (β*_mean, LINE-1_*) in pooled cfDNA samples from healthy individuals and pooled cfDNA samples from CRC patients in GSE186381. The gray line represents β*_mean, OS_* in adjacent normal tissue from the TCGA study.

**Fig. S11.** Comparison of mean β-value across OpenSea probes (β*_mean, OS_*) in pooled cfDNA samples from healthy individuals, pooled cfDNA samples from patients with advanced adenoma, and pooled cfDNA samples from CRC patients in GSE110185. The gray line represents β*_mean, OS_* in adjacent normal tissue from the TCGA study.

**References**

1.	Díez-Villanueva A, Sanz-Pamplona R, Carreras-Torres R, Moratalla-Navarro F, Alonso MH, Paré-Brunet L, et al. DNA methylation events in transcription factors and gene expression changes in colon cancer. Epigenomics. 2020 Sep;12(18):1593–610. doi:10.2217/epi-2020-0029 PubMed PMID: 32957849.

2.	Naumov VA, Generozov EV, Zaharjevskaya NB, Matushkina DS, Larin AK, Chernyshov SV, et al. Genome-scale analysis of DNA methylation in colorectal cancer using Infinium HumanMethylation450 BeadChips. Epigenetics. 2013 Sep;8(9):921–34. doi:10.4161/epi.25577 PubMed PMID: 23867710; PubMed Central PMCID: PMC3883769.

3.	Luo Y, Wong CJ, Kaz AM, Dzieciatkowski S, Carter KT, Morris SM, et al. Differences in DNA methylation signatures reveal multiple pathways of progression from adenoma to colorectal cancer. Gastroenterology. 2014 Aug;147(2):418-429.e8. doi:10.1053/j.gastro.2014.04.039 PubMed PMID: 24793120; PubMed Central PMCID: PMC4107146.

4.	Schultz BM, Ayeotan O, Rana JN, Litwin S, Basickes S, Sapienza C, et al. Abnormal epigenetic aging of epigenetic outliers in normal colon mucosa from colorectal cancer patients. Sci Rep. 2026 Jan 7;16(1):4022. doi:10.1038/s41598-025-34035-x

5.	Wang C. Epigenome analysis of normal and colorectal cancer (CRC) samples [Internet]. Available from: https://www.ncbi.nlm.nih.gov/geo/query/acc.cgi?acc=GSE240324

6.	Kennedy DW, White NM, Benton MC, Fox A, Scott RJ, Griffiths LR, et al. Critical evaluation of linear regression models for cell-subtype specific methylation signal from mixed blood cell DNA. PLOS ONE. 2018 Dec 20;13(12):e0208915. doi:10.1371/journal.pone.0208915

7.	Liu Y, Aryee MJ, Padyukov L, Fallin MD, Hesselberg E, Runarsson A, et al. Epigenome-wide association data implicate DNA methylation as an intermediary of genetic risk in rheumatoid arthritis. Nat Biotechnol. 2013 Feb;31(2):142–7. doi:10.1038/nbt.2487 PubMed PMID: 23334450; PubMed Central PMCID: PMC3598632.

8.	Gallardo-Gómez M, Moran S, Páez de la Cadena M, Martínez-Zorzano VS, Rodríguez-Berrocal FJ, Rodríguez-Girondo M, et al. A new approach to epigenome-wide discovery of non-invasive methylation biomarkers for colorectal cancer screening in circulating cell-free DNA using pooled samples. Clin Epigenetics. 2018 Apr 16;10(1):53. doi:10.1186/s13148-018-0487-y

9.	Gallardo-Gómez M, Rodríguez-Girondo M, Planell N, Moran S, Bujanda L, Etxart A, et al. Serum methylation of GALNT9, UPF3A, WARS, and LDB2 as noninvasive biomarkers for the early detection of colorectal cancer and advanced adenomas. Clin Epigenetics. 2023 Oct 4;15(1):157. doi:10.1186/s13148-023-01570-1

10.	Moss J, Magenheim J, Neiman D, Zemmour H, Loyfer N, Korach A, et al. Comprehensive human cell-type methylation atlas reveals origins of circulating cell-free DNA in health and disease. Nat Commun. 2018 Nov 29;9(1):5068. doi:10.1038/s41467-018-07466-6
